# Comparison of diet and exercise on cardiometabolic factors in young adults with overweight/obesity: multiomics analysis and gut microbiota prediction, a randomized controlled trial

**DOI:** 10.1002/mco2.70044

**Published:** 2025-01-12

**Authors:** Zongyu Lin, Tianze Li, Fenglian Huang, Miao Wu, Lewei Zhu, Yueqin Zhou, Ying‐An Ming, Zhijun Lu, Wei Peng, Fei Gao, Yanna Zhu

**Affiliations:** ^1^ Department of Maternal and Child Health School of Public Health Sun Yat‐sen University Guangzhou China; ^2^ Department of Physical Education Sun Yat‐sen University Guangzhou China; ^3^ Agricultural Genomics Institute at Shenzhen Chinese Academy of Agricultural Sciences Shenzhen China; ^4^ Guangdong Provincial Key Laboratory of Food Nutrition and Health Guangzhou China

**Keywords:** aerobic exercise, cardiometabolic factors, fiber‐rich diet, gut microbiota, prediction, young adults

## Abstract

The optimal strategy for improving cardiometabolic factors (CMFs) in young obese individuals through diet and exercise remains unclear, as do the potential mechanisms. We conducted an 8‐week randomized controlled trial to compare the effects of different interventions in youth with overweight/obesity. Gut microbes and serum metabolites were examined to identify regulating mechanisms. A total of 129 undergraduates were randomly assigned to fiber‐rich (FR) diet, rope‐skipping (RS), combined FR–RS and control groups. The results showed that single interventions were as effective as combined interventions in improving weight, waist circumference, body fat, and lipid profile compared with control group. Notably, the FR group further reduced low‐density lipoprotein (LDL‐C) and uric acid (UA) (all *p* < 0.05). Mediation analysis revealed four gut microbiota–metabolite–host axes in improving CMFs. Additionally, we used machine learning algorithms to further predict individual responses based on baseline gut microbiota composition, with specific microbial genera guiding targeted intervention selection. In conclusion, FR diet and/or RS were effective in improving CMFs, with the FR diet particular effectiveness in reducing LDL‐C and UA levels. These benefits may drive by gut microbiome–metabolite–host interactions. Moreover, the predictability of gut microbiota composition supports making targeted decisions in selecting interventions. Trial Registration: NCT04834687.

## Graphical abstract

An 8‐week randomized controlled trial were conducted to compare the effects of fiber‐rich diet (FR) and/or rope‐skipping (RS) on cardiometabolic factors (CMFs) in youth with overweight/obesity. The results showed that FR and/or RS were all effective in improving CMFs. These benefits may drive by gut microbiome–metabolite–host interactions. Moreover, the predictability of gut microbiota composition supports making targeted decisions in selecting interventions.

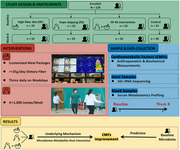

## 1 INTRODUCTION

Currently, cardiovascular diseases (CVDs) remain the leading cause of death globally, accounting for 32% of all deaths in 2019.^1^ Notably, this proportion reaches 40% in China, making it the nation with the highest global CVD burden.^1^, ^2^ The abnormal levels of cardiometabolic factors (CMFs) among young individuals, such as obesity, lipid irregularities, and high blood glucose levels, have also significantly increased over the past two decades.^3^ This trend has raised concerns about the increased risk of CVDs in later life for this population.^3^, ^4^, ^5^ Thus, it is of considerable importance to implement effective interventions to improve CMFs levels as early as possible.

Although both fiber‐rich (FR) diet and aerobic exercise (AE) have demonstrated benefits for weight control,^6^, ^7^ improvement in lipid metabolism,^8^, ^9^ and reduction of fasting plasma glucose (FPG),^8^, ^10^ there is still a lack of sufficient research evidence to determine the optimal intervention strategy for each indicator due to the complexity and diversity of CMFs. Moreover, a single measure is unlikely to improve all CMFs. For example, while certain interventions may be highly effective in controlling weight, they may have minimal impact on other indicators. Additionally, the mechanisms through which FR and AE interventions improve CMF levels involve multiple metabolic pathways that are not yet fully understood. Therefore, further research is needed to identify the most effective intervention strategies based on different CMFs, providing stronger guidance for personalized health management and clinical applications.

A series of studies have reported that both FR diet and AE interventions alone can regulate CMF levels through the gut microbiota and its metabolites.^11^, ^12^, ^13^, ^14^, ^15^ Dietary fiber serves as a substrate for intestinal flora, improving gut microbiota diversity and richness^11^ and leading to the production of short‐chain fatty acids (SCFAs).^12^, ^13^ At the same time, AE has been showed to increase gut microbiota diversity^14^ and restore a healthy bacterial composition.^15^ However, the underlying mechanisms and the variations in how FR diets and AE benefit CMFs still require further elucidation.

Another obstacle in selecting the optimal intervention is the variability in individual responses, which leads some participants to benefit more from the same intervention.^16^ This variability also makes it nearly impossible to achieve comprehensive improvements in all CMFs across individuals with a single intervention. Growing evidence suggested that highly individualized nature of the gut microbiome plays a key role in these interindividual differences in responses.^17^, ^18^ Consequently, to better understand the distinctions between responders and nonresponders or low responders and to identify effective intervention strategies, it is essential to analyze microbiome composition at the individual level.

Therefore, a randomized controlled trial (RCT) was conducted among youth to investigate the effects of different interventions and identify appropriate strategy for improving specific CMFs at the population level. Additionally, we explored the potential roles of gut microbiota and serum metabolites in mediating CMFs under different interventions. Finally, we hypothesized that the baseline microbiota composition could predict individual improvements in CMFs following various interventions, enabling the identification of intervention‐specific genera as biomarkers to assist in selecting optimal individual strategies.

## 2 RESULTS

### 2.1 Study overview and participant characteristics at baseline

**FIGURE 1:**
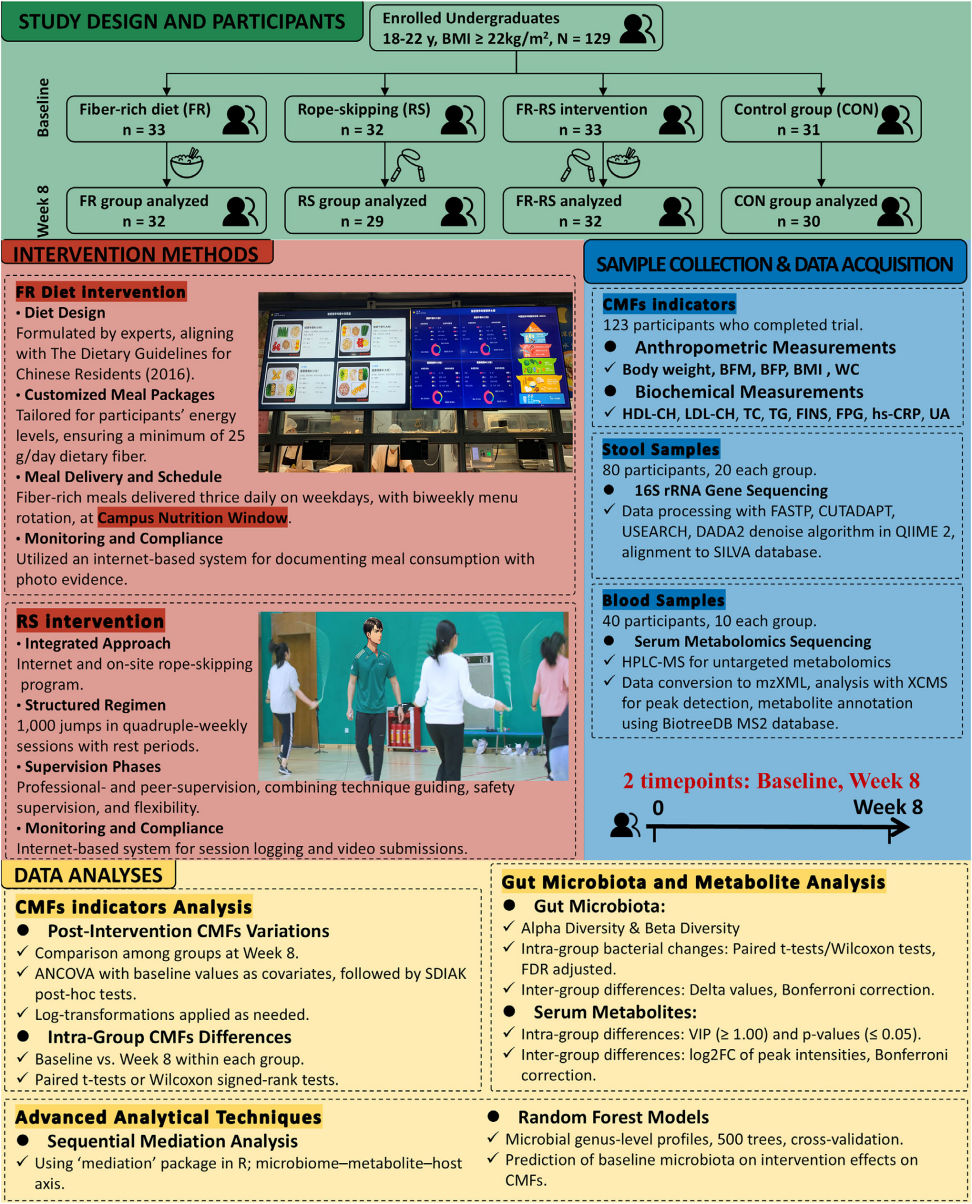
Study design. ANCOVA, analysis of covariance; BFM, body fat mass; BMI, body mass index; BFP, body fat percentage; CMFs, cardiometabolic factors; CON, control group; FR, fiber‐rich diet group; FR–RS, fiber‐rich diet and rope‐skipping group; FINS, fasting insulin; FPG, fasting plasma glucose; HDL‐C, high‐density lipoprotein cholesterol; hs‐CRP, high‐sensitivity C‐reactive protein; LDL‐C, low‐density lipoprotein cholesterol; Log2FC, the log2 transformed fold changes of the peak intensity of individual metabolites of 8‐week/baseline; RS, rope‐skipping group; TC, total cholesterol; TG, triglyceride; UA, uric acid; WC, waist circumference.

A total of 129 undergraduates with overweight/obesity were enrolled in this parallel‐designed RCT. Participants were stratified by sex and weight, then randomly assigned to one of four groups. Finally, 123 participants completed both the intervention and the follow‐up (see Figures 1 and S1 for details). No participants reported any adverse events. The participants were aged between 18 and 21, and 44.7% were male. Baseline characteristics of the participants were shown in Table 1. Participants in rope‐skipping (RS) and FR and RS (FR–RS) group improved physical activity intensity, and those in FR and FR–RS group increased dietary fiber intake (see Table S1 for details).

**TABLE 1:** Baseline characteristics of the participants.

Characteristic	RS	FR	FR–RS	Control	*p* value
*n*	29	32	32	30	
Male	13 (44.8)	14 (43.8)	12 (40.6)	15 (50.0)	0.785
Age, y	18.1 ± 0.8	18.1 ± 0.6	18.2 ± 1.4	19.3 ± 0.9	<0.050
BMI, kg/m^2^	25.0 ± 2.7	25.4 ± 3.1	25.3 ± 3.6	24.8 ± 2.9	0.862
Mode of delivery					0.277
Vaginal delivery	15 (53.6)	14 (45.2)	21 (67.7)	19 (63.3)	
Cesarean delivery	13 (46.4)	17 (54.8)	10 (32.3)	11 (36.7)	
Paternal educational level					0.116
Junior high school and below	5 (17.9)	7 (22.6)	5 (16.1)	5 (16.7)	
High school to junior college	14 (50.0)	8 (25.8)	20 (64.5)	14 (46.7)	
Bachelor's degree and above	9 (32.1)	16 (51.6)	6 (19.4)	11 (36.7)	
Maternal educational level					0.126
Junior high school and below	6 (21.4)	5 (16.1)	13 (41.9)	6 (20.0)	
High school to junior college	14 (50.0)	14 (45.2)	12 (38.7)	10 (33.3)	
Bachelor's degree and above	8 (28.6)	12 (38.7)	6 (19.4)	14 (46.7)	
Monthly household income, RMB/person					0.793
<5000	12 (46.2)	14 (53.9)	17 (58.6)	15 (57.7)	
≥5000	14 (53.9)	12 (46.2)	12 (41.34)	11 (42.3)	
Dietary intake					
Energy, kcal/d	1601.5 (1293.0, 1846.0)	1704.5 (1391.0, 2160.0)	1755.0 (1536.0, 2219.0)	1648.5 (1385.0, 1801.5)	0.232
Protein, g/d	68.7 (55.6, 85.1)	67.8 (55.9, 93.4)	75.0 (56.6, 89.2)	58.8 (50.5, 79.5)	0.294
Dietary fat, g/d	57.8 (44.4, 65.6)	68.1 (45.2, 78.9)	70.3 (54.5, 87.8)	63.5 (54.3, 70.2)	0.080
Carbohydrates, g/d	205.7 (149.9, 246.6)	219.5 (189.6, 271.8)	205.0 (158.2, 269.3)	188.1 (160.5, 236.0)	0.370
Dietary fiber, g/d	8.4 (5.2, 13.6)	9.7 (6.0, 12.8)	7.6 (5.2, 10.0)	6.6 (5.3, 9.6)	0.451
Physical activity intensity, (METs/minutes/week)	1693.0 (1035.0, 1910.0)	1639.0 (924.0, 2332.0)	1702.0 (1266.2, 2621.5)	1794.0 (1024.0, 2506.0)	0.560
Sedentary time, h/d	7.5 (6.0, 9.0)	7.0 (5.0, 9.0)	8.5 (6.5, 10.0)	8.0 (6.0, 10.0)	0.755
Outdoor time, h/d					0.959
<2	26 (92.9)	28 (90.3)	28 (90.3)	28 (93.3)	
≥2	2 (7.1)	3 (9.7)	3 (9.7)	2 (6.7)	

*Note*: Data are presented as mean ± SD, median (IQR), or *n* (%), *p* values among groups were determined by analysis of covariance, Kruskal–Wallis test, or chi‐square test.

Abbreviations: FR, fiber‐rich diet group; FR–RS, fiber‐rich diet and rope‐skipping group; IQR, interquartile range; METs, metabolic equivalents task; RS, rope‐skipping group.

### 2.2 Individual and combined interventions all significantly improved CMFs

#### 2.2.1 Population level effects: combined interventions were not superior to individual

After 8 weeks intervention, significant improvements were observed in body composition indicators, including weight, waist circumference (WC), body fat mass (BFM), and body fat percentage (BFP), in the FR–RS group, RS group, and FR group compared with the control group (all *p* < 0.05). However, only FR and FR–RS interventions reduced body mass index (BMI) and triglyceride (TG) compared with the control group (all *p* < 0.05). Additionally, the 8‐week FR diet resulted in significant decreases in serum total cholesterol (TC), uric acid (UA), and low‐density lipoprotein cholesterol (LDL‐C) levels (all *p* < 0.01), as detailed in Table 2.

**TABLE 2:** Comparing the effects of RS, FR, and FR–RS interventions with control on CMFs in youth with overweight/obesity.

Variables	RS (*n* = 29)	FR (*n* = 32)	FR–RS (*n* = 32)	Control (*n* = 30)	*p* value
Body weight, kg					
Baseline^a^	69.12 (10.51)	71.93 (11.62)	71.92 (16.34)	71.64 (13.01)	0.811
Change^a^	−1.5 (1.91)^c^	−1.68 (2.40)^c^	−1.93 (3.24)^c^	0.21 (1.62)	**0.003**
8‐week adjusted^b^	69.56 (68.73, 70.39)^**^	69.56 (68.77, 70.34)^**^	69.31 (68.53, 70.10)^**^	71.43 (70.62, 72.25)	**0.001**
WC, cm					
Baseline^a^	82.88 (8.75)	83.04 (10.17)	83.03 (11.36)	80.88 (9.47)	0.800
Change^a^	−1.99 (3.99)^c^	−1.19 (3.59)	−2.59 (4.23)^c^	1.55 (3.05)	**<0.001**
8‐week adjusted^b^	80.54 (79.28, 81.81)^**^	81.36 (80.16, 82.57)^*^	79.96 (78.76, 81.17)^**^	83.79 (82.54, 85.04)	**< 0.001**
BFM, kg					
Baseline^a^	20.28 (5.23)	21.55 (5.49)	21.82 (7.16)	20.12 (6.01)	0.593
Change^a^	−1.70 (1.26)^c^	−1.77 (1.88)^c^	−1.94 (2.39)^c^	−0.23 (1.18)	**0.001**
8‐week adjusted^b^	19.28 (18.63, 19.93)^**^	19.2 (18.58, 19.82)^**^	19.02 (18.4, 19.65)^**^	20.75 (20.11, 21.40)	**0.001**
BFP, %					
Baseline^a^	29.46 (6.32)	30.11 (6.26)	30.32 (5.91)	28.35 (7.00)	0.622
Change^a^	−1.95 (1.71)^c^	−1.9 (1.83)^c^	−2.14 (2.24)^c^	−0.41 (1.10)	**0.001**
8‐week adjusted^b^	27.64 (27.01, 28.28)^**^	27.64 (27.04, 28.25)^**^	27.39 (26.78, 27.99)^**^	29.26 (28.63, 29.88)	**<0.001**
BMI, kg/m^2^					
Baseline^a^	25.02 (2.72)	25.42 (3.07)	25.25 (3.56)	24.78 (2.93)	0.862
Change^a^	−0.55 (0.69)^c^	−0.71 (0.81)^c^	−0.68 (1.16)^c^	0.01 (0.57)	**0.003**
8‐week adjusted^b^	24.57 (24.27, 24.87)	24.44 (24.15, 24.72)^**^	24.45 (24.16, 24.74)^**^	25.12 (24.82, 25.42)	**0.004**
TC, mmol/L					
Baseline^a^	4.32 (0.53)	4.34 (0.65)	4.47 (0.50)	4.41 (0.69)	0.754
Change^a^	−0.24 (0.35)^c^	−0.44 (0.39)^c^	−0.24 (0.59)^c^	0.00 (0.34)	**0.002**
8‐week adjusted^b^	4.13 (3.98, 4.28)	3.94 (3.79, 4.08)^**^	4.17 (4.02, 4.31)	4.39 (4.24, 4.53)	**0.001**
TG, mmol/L					
Baseline^a^	0.97 (0.31)	0.98 (0.62)	1.06 (0.46)	1.02 (0.50)	0.872
Change^a^	−0.05 (0.29)	−0.19 (0.35)^c^	−0.18 (0.41)^c^	0.04 (0.29)	**0.023**
8‐week adjusted^b^	0.94 (0.84, 1.04)	0.8 (0.71, 0.89)^**^	0.85 (0.76, 0.95)^*^	1.06 (0.96, 1.16)	**0.002**
LDL‐C, mmol/L					
Baseline^a^	2.53 (0.52)	2.54 (0.55)	2.6 (0.50)	2.65 (0.57)	0.808
Change^a^	−0.20 (0.34)^c^	−0.35 (0.32)^c^	−0.15 (0.49)	−0.01 (0.28)	**0.006**
8‐week adjusted^b^	2.37 (2.24, 2.50)	2.22 (2.10, 2.35)^**^	2.43 (2.31, 2.56)	2.58 (2.45, 2.71)	**0.001**
HDL‐C, mmol/L					
Baseline^a^	1.42 (0.25)	1.42 (0.29)	1.46 (0.34)	1.37 (0.32)	0.677
Change^a^	−0.01 (0.12)	−0.01 (0.14)	0.04 (0.23)	0 (0.13)	0.658
8‐week adjusted^b^	1.41 (1.35, 1.47)	1.41 (1.35, 1.46)	1.46 (1.40, 1.51)	1.41 (1.35, 1.47)	0.528
FPG, mmol/L					
Baseline^a^	4.40 (0.32)	4.36 (0.49)	4.58 (0.35)	4.37 (0.47)	0.147
Change^a^	0.12 (0.27)^c^	0.14 (0.61)	−0.01 (0.42)	0.16 (0.41)^c^	0.431
8‐week adjusted^b^	4.53 (4.39, 4.66)	4.53 (4.40, 4.66)	4.51 (4.38, 4.64)	4.55 (4.42, 4.68)	0.979
FINs, µU/mL					
Baseline^a^	9.66 (6.44)	10.28 (6.38)	11.99 (6.03)	10.95 (6.55)	0.521
Change^a^	1.6 (5.35)	0.75 (8.27)	2.27 (18.26)	2.98 (6.81)	0.875
8‐week adjusted^b^	11.91 (7.91, 15.90)	11.31 (7.52, 15.10)	13.51 (9.70, 17.32)	13.81 (9.89, 17.72)	0.762
hs‐CRP, mg/L					
Baseline^a^	1.07 (1.67)	0.93 (1.16)	1.27 (1.63)	1.48 (3.32)	0.757
Change^a^	−0.44 (1.91)	0.06 (2.18)	−0.36 (1.72)	−0.63 (3.3)	0.702
8‐week adjusted^b^	0.64 (0.13, 1.14)	1.01 (0.53, 1.49)	0.91 (0.43, 1.39)	0.82 (0.33, 1.32)	0.745
UA, µmol/L					
Baseline^a^	422.79 (100.66)	429.59 (113.03)	419.81 (134.33)	394.17 (85.88)	0.617
Change^a^	−29.66 (59.86)^c^	−59.88 (50.95)^c^	−43.00 (116.69)^c^	5.80 (54.55)	**0.008**
8‐week adjusted^b^	389.48 (366.29, 412.67)	361.91 (339.8, 384.02)^**^	374.98 (352.9, 397.05)	413.79 (390.88, 436.71)	**0.012**

Comparison of 8‐week effects adjusting for baseline between intervention groups and control groups, assessed by Bonferroni corrected *t*‐test: *p < 0.05, **p < 0.01.

Abbreviations: BFM, body fat mass; BFP, body fat percentage; BMI, body mass index; CMFs, cardiometabolic factors; FINS, fasting insulin; FPG, fasting plasma glucose; FR, fiber‐rich diet group; FR–RS, fiber‐rich diet and rope‐skipping group; HDL‐C, high‐density lipoprotein cholesterol; hs‐CRP, high‐sensitivity C‐reactive protein; LDL‐C, low‐density lipoprotein cholesterol; RS, rope‐skipping group; TC, total cholesterol; TG, triglyceride; UA, uric acid; WC, waist circumference.

^a^ Data are presented as mean (SD), *p* values among groups were obtained by analysis of variance.

^b^ Data are presented as mean (95% CI), adjusted for baseline measurements, *p* values among groups were obtained by analysis of covariance with baseline as a covariate.

^c^ *p* < 0.05, within‐group differences (baseline vs. 8 weeks) analyzed by paired sample *t*‐test or Wilcoxon signed rank test.

Most CMFs showed improvement with at least one of the interventions, except for high‐sensitivity C‐reactive protein (hs‐CRP), FPG, and fasting insulin (FINS). Therefore, further comparisons were conducted to identify the most effective intervention when multiple interventions were efficacious. Regrettably, none of the interventions demonstrated a significant advantage in improving any of the same CMFs (all *p* value > 0.05; Table S2). Although the FR–RS intervention trended to show better outcomes for specific CMFs, it did not achieve statistical significance when compared with individual interventions.

#### 2.2.2 Individual level effects: variable responses to the same intervention

Our results indicate that while the FR, RS, and FR–RS interventions all led to significant improvements in the same CMFs at the population level, there were notable differences in individual responses to the same intervention (Figure S2). Furthermore, under the same intervention, some individuals showed improvement in one CMF but not in others, suggesting that the effects of these interventions on each CMF are highly personalized and exhibit considerable variability.

### 2.3 The potential role of gut microbiota and serum metabolites on CMFs improvement

#### 2.3.1 Changes of diversity and structure of gut microbiota induced by different interventions

**FIGURE 2:**
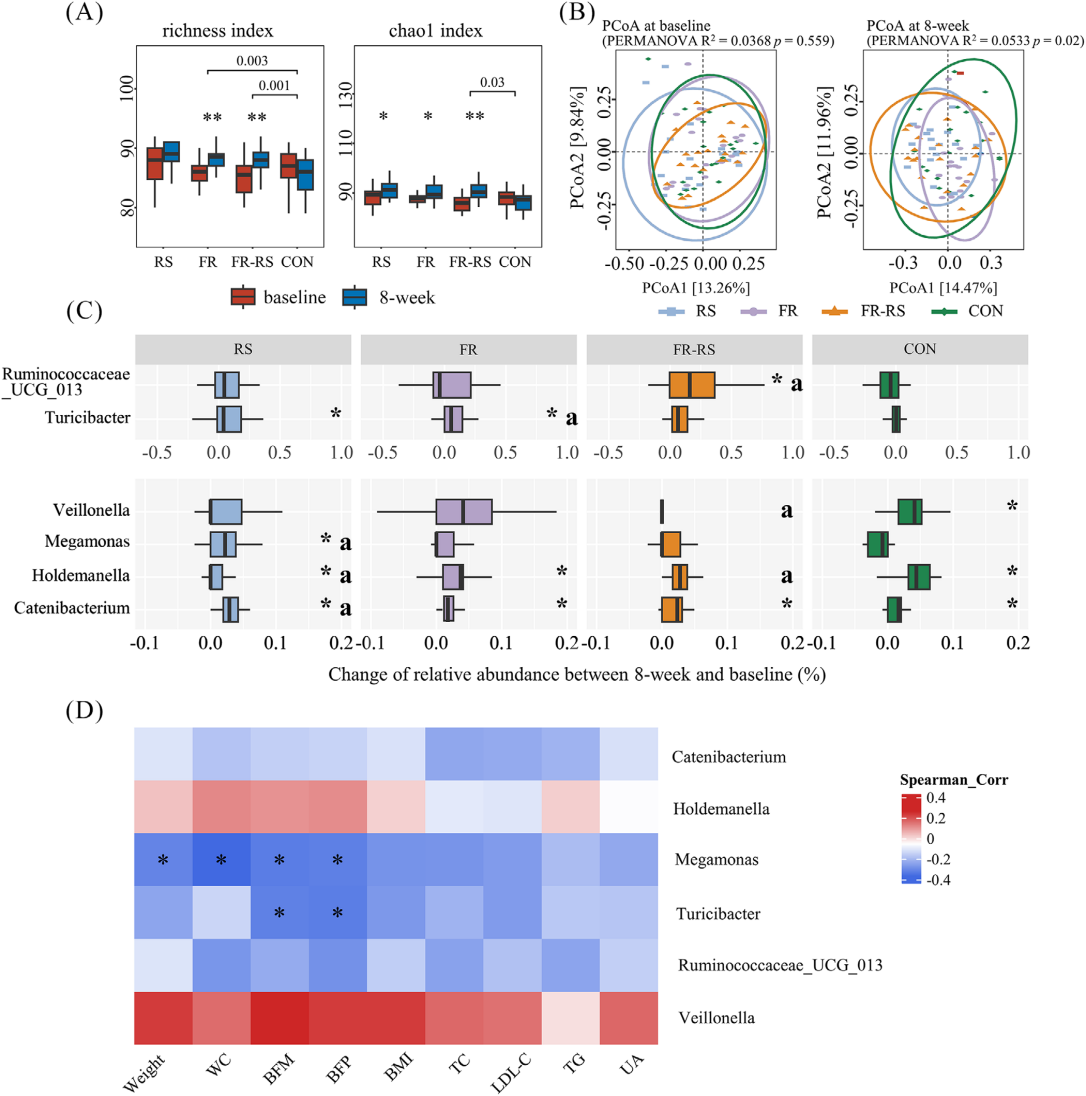
Effects of fiber‐rich diet and rope‐skipping interventions on gut microbiota. (A) Fecal microbial α‐diversity at OTU level before and after 8‐week intervention. (B) Fecal microbial β‐diversity at OTU level before and after 8‐week intervention. (C) Changes of relative abundance of gut microbiota at genus level. a indicates a significantly statistic difference compared with control group, *p* ≤ 0.05. (D) Association between changes of microbial relative abundance at genus level and CMFs. * Indicates a significant association evaluated by spearman correcting for FDR: *q_FDR ≤ 0.05. RS, rope‐kipping group; FR, fiber‐rich group; FR–RS, fiber‐rich diet and rope‐kipping group; CMFs, cardiometabolic factors; FDR, false discovery rate; WC, waist circumference; BFM, body fat mass; BFP, body fat percentage; BMI, body mass index; LDL‐C, low‐density lipoprotein cholesterol; TC, total cholesterol; TG, triglyceride; UA, uric acid.

Following the 8‐week intervention, a significant increase in gut microbial richness was observed in both the FR and FR–RS groups in comparison with the control group (all *p* < 0.01). The Chao1 index increase in the FR–RS group was also significantly greater than in the control group (*p* < 0.05) (Figure 2A). The β‐diversity analysis indicated similar gut microbiota across the four groups before intervention, but significant differences emerged postintervention (*p* < 0.05) (Figure 2B). Specifically, FR–RS group exhibited a significant change following the intervention (*p* < 0.05) (Figure S3).

At the phylum level, we found that the relative abundance of *Actinobacteria* significantly increased in the RS group compared with the baseline. In contrast, the FR–RS intervention decreased the relative abundance of *Proteobacteria* (Figure S4). At the genus level, compared with the control group, the RS intervention increased the relative abundance of *Megamonas*, *Holdemanella*, and *Catenibacterium*, while the FR intervention resulted in a higher abundance of *Turicibacter*. The FR–RS intervention was associated with an increased abundance of *Ruminococcaceae_UCG‐013* (*p* < 0.05) (Figure 2C).

The results of the correlation analysis revealed an inverse association between the changes in the relative abundance of *Megamonas* and changes in body weight, WC, BFM, and BFP (all *p* < 0.05, q_ False Discovery Rate (FDR) < 0.05). Similarly, a negative correlation was noted between changes in *Turicibacter* and reductions in BFM and BFP (all *p* < 0.05, q_FDR < 0.05) (Figure 2D).

#### 2.3.2 Changes of serum metabolites induced by different interventions

**FIGURE 3:**
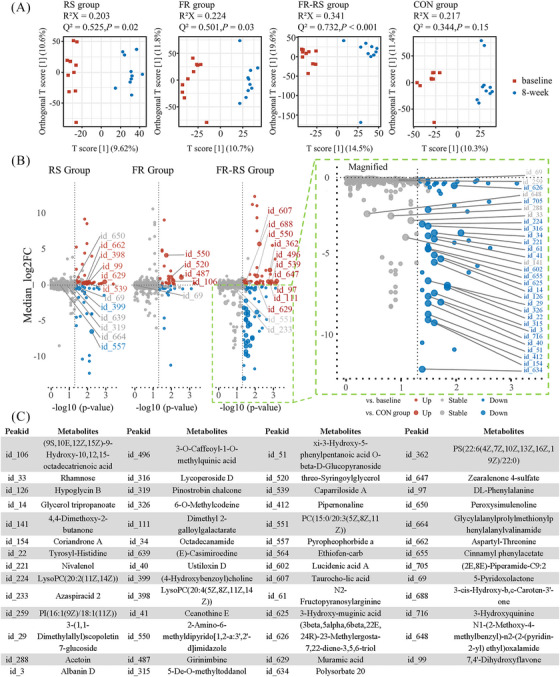
Effects of FR diet and AE interventions on serum metabolites. (A) OPLS‐DA score plot at positive ion mode of each group before and after 8‐week intervention. (B) Significantly changed serum metabolites at positive ion mode of each intervention group after 8‐week intervention. Colored dots indicate significance referring to baseline. Larger dots with labeled id indicate significance referring to control group. (C) Taxonomy information of labeled metabolites. AE, aerobic exercise; RS, rope‐skipping group; FR, fiber‐rich diet group; FR–RS, fiber‐rich diet and rope‐skipping group.

Following the 8‐week intervention, notable changes were detected in the composition of serum metabolites in the RS, FR, and FR–RS groups under the positive ion mode (*p* < 0.05). This was in stark contrast to the control group, which exhibited minimal changes (*p* = 0.15) (Figure 3A). However, no notable changes were observed in negative ion mode across any of the groups (Figure S5).

When comparing individual metabolites to baseline, the RS group showed an upregulation of 65 metabolites and a downregulation of 38. The FR diet resulted in the upregulation of 49 metabolites and downregulation of 38. The most significant changes were observed in the FR–RS group, with 67 metabolites upregulated and a notable 90 downregulated. (Figures 3B and S6).

Compared with the control group, the RS intervention led to a significant upregulation of 11 metabolites, while four metabolites, such as [4‐Hydroxybenzoyl]choline, were significantly downregulated. Similarly, the FR diet resulted in the upregulation of five metabolites, while four metabolites, were downregulated (Figures 3B and S6).

For the FR–RS intervention, participants exhibited a significant upregulation of 16 metabolites, primarily categorized under the super classes of organic oxygen compounds, lipids and lipid‐like molecules, and organic acids and derivatives. A substantial downregulation was also observed in the FR–RS group, with 34 metabolites showing a decrease compared with the control group. The majority of these downregulated metabolites belonged to the super classes of lipids and lipid‐like molecules, as well as organic acids and derivatives and organo heterocyclic compounds (Figures 3B,C and S6).

To gain a deeper understanding of the metabolic effects of different interventions, we performed pathway and enrichment analyses using the KEGG database (Figure S7). The KEGG pathway analysis of serum metabolites revealed distinct metabolic pathways enriched in the FR, RS, and FR–RS groups. In the FR group, the primary enriched pathways were “unsaturated fatty acid biosynthesis” and “arachidonic acid metabolism.” These findings suggest that the FR intervention may improve lipid metabolism by influencing fatty acid metabolic pathways.

In the RS group, the enriched metabolic pathways included “pantothenate and CoA biosynthesis” and “beta‐alanine metabolism.” These pathways are related to energy metabolism and coenzyme A synthesis. In the FR–RS group, pathway enrichment analysis revealed broader metabolic changes, covering pathways such as “taurine and hypotaurine metabolism,” “primary bile acid biosynthesis,” and “phenylalanine, tyrosine, and tryptophan biosynthesis”. Given the sufficient number of differential metabolites in the FR–RS group for further analysis, pathway enrichment revealed that the “phenylalanine, tyrosine, and tryptophan biosynthesis” pathway had the highest impact, highlighting its critical role under the combined intervention. Additionally, the enrichment of the “primary bile acid biosynthesis” pathway points to potential regulation of bile acid metabolism, contributing to improved lipid metabolism (Figure S8). Overall, these enriched pathways provide new insights into how the combined intervention synergistically regulates multiple metabolic pathways to improve CMFs.

#### 2.3.3 Sequential mediation analyses of gut microbiota, serum metabolites, and CMFs

**FIGURE 4:**
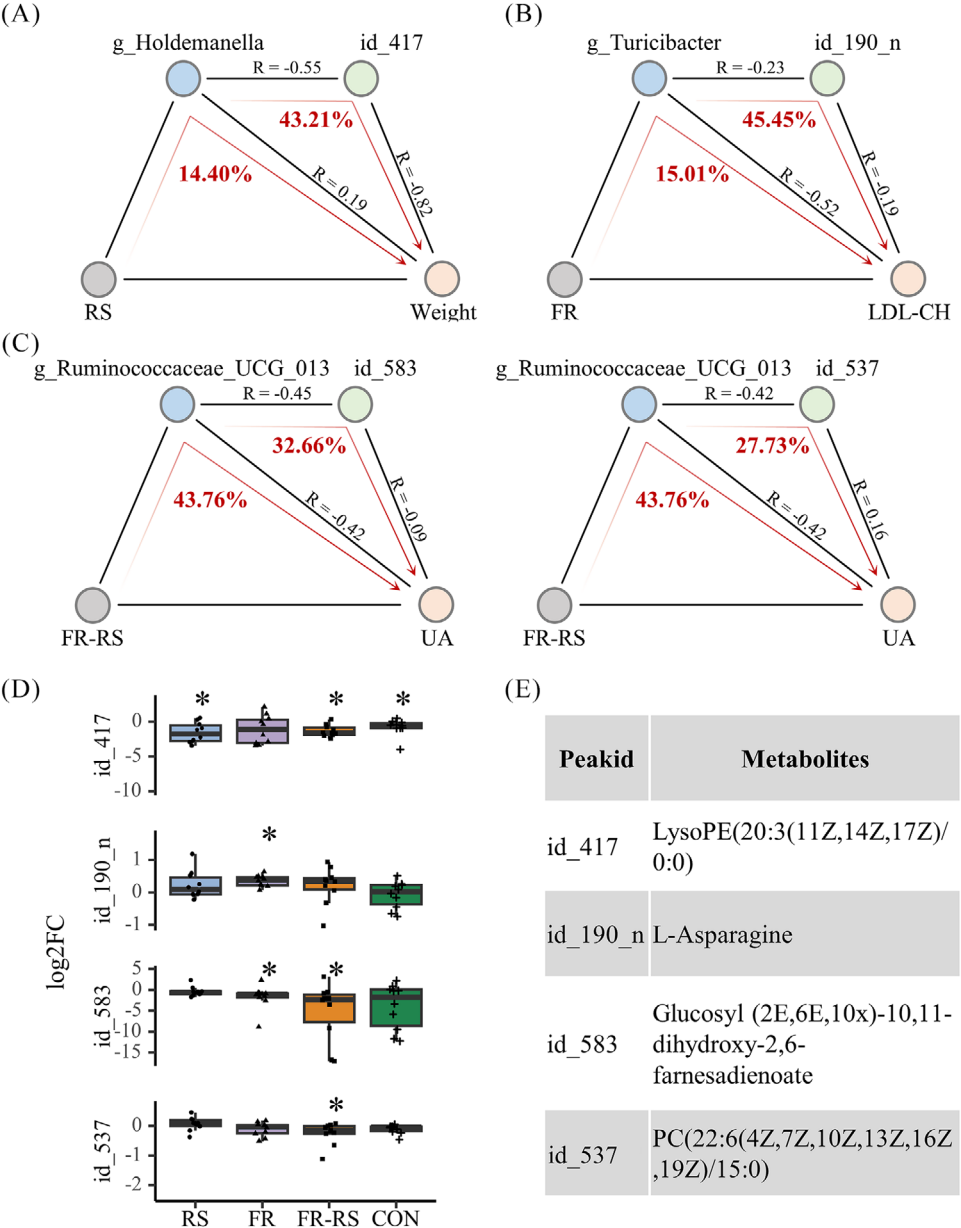
Sequential analyses of multiomics data along the microbiome–metabolite–host axis. (A) RS group; (B) FR group; (C) FR–RS group. Colored node represents intervention methods (light gray), a gut bacterium (light blue), a serum metabolite (light green) or a CMFs indicator (light pink). The pathways of mediation analysis are indicated with red arrows. The proportions (%) of mediation effects are indicated in red numbers. (D) Log2FC of individual metabolites. *: significance between baseline versus 8 week. (E) Queries of labeled metabolites. RS, rope‐skipping group; FR, fiber‐rich diet group; FR–RS, fiber‐rich diet and rope‐kipping group; CMFs, cardiometabolic factors; LDL‐C, low‐density lipoprotein cholesterol; UA, uric acid; Log2FC, the log2 transformed fold changes of the peak intensity of individual metabolites of 8‐week/baseline.

The sequential mediation analyses illuminate compelling insights into the impacts of RS, FR, and FR–RS interventions on CMFs through the microbiome–metabolite–host axis. The effect of RS intervention on improving body weight is partially mediated by the genus *Holdemanella* (proportion‐mediated: 14.40%, *p* value for average causal mediation effects [ACME]: 0.022). Concurrently, the downstream effect of the *Holdemanella* genus on body weight is further mediated by the metabolite LysoPE(20:3(11Z,14Z,17Z)/0:0) (proportion‐mediated: 43.21%; suggestive trend, *p* values for ACME: 0.082, respectively) (Figure 4A). This indicated that part of the intervention's effect occurs through modulation of *Holdemanella*, with further downstream effects on body weight potentially mediated by metabolite LysoPE(20:3).

Regarding the FR intervention, it has been noted that the amelioration of LDL‐C is partially mediated by the genus *Turicibacter* (proportion‐mediated: 15.01%; suggestive trend, *p* value for ACME: 0.086). Subsequently, the effect of the genus *Turicibacter* on LDL‐C is also partially mediated by the metabolite l‐asparagine (proportion‐mediated: 45.45%; suggestive trend, *p* value for ACME: 0.092) (Figure 4B). Finally, the FR–RS intervention exerts the effect on UA through the partial mediation of *Ruminococcaceae_UCG_013* (proportion‐mediated: 43.76%, *p* value for ACME: 0.022). Simultaneously, the effect of *Ruminococcaceae_UCG_013* on UA is mediated through the metabolites glucosyl (2E, 6E, 10×)‐10,11‐dihydroxy‐2,6‐farnesadienoate and PC (22:6(4Z,7Z,10Z,13Z,16Z,19Z)/15:0) (proportion‐mediated: 32.66 and 27.73%; *p* values for ACME: 0.018 and 0.094 with suggestive trend, respectively) (Figure 4C).

### 2.4 Gut microbiota predicts individual responsiveness to different intervention

**FIGURE 5:**
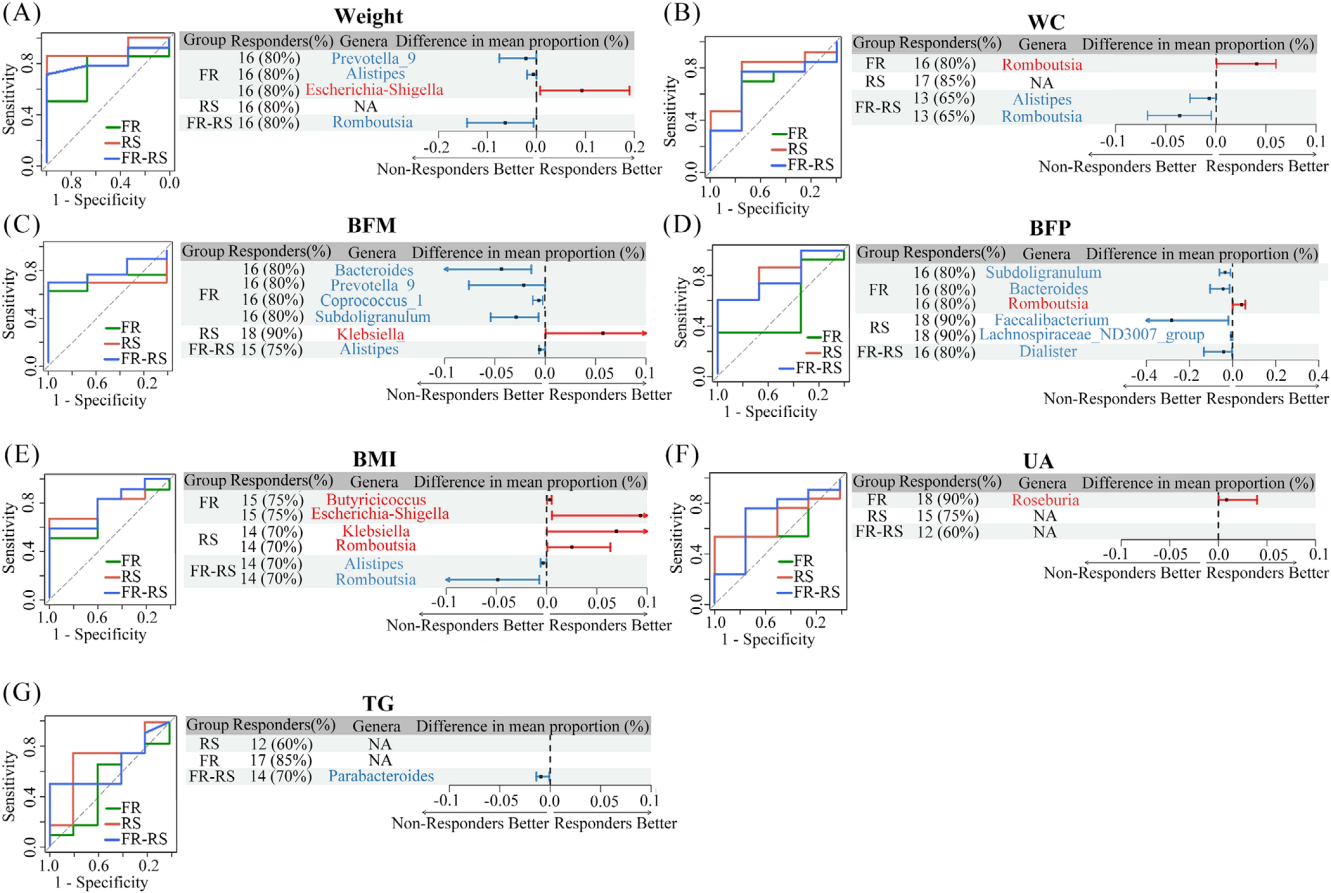
Baseline gut microbiota predicting the improvement of body composition, UA, and TG upon different interventions. On the left are the ROC curves of predicting CMFs responders upon interventions based on the baseline genus abundance by random forest models. On the right are the numbers (%) of the responders, the differential genera (red, higher in responders; blue, lower in responders) among top 10 general contributing the modelling and their mean abundance difference between responders and nonresponders. RS, rope‐kipping group; FR, fiber‐rich group; FR–RS, fiber‐rich diet and rope‐kipping group; CMFs, cardiometabolic factors; WC, waist circumference; BFM, body fat mass; BFP, body fat percentage; BMI, body mass index; TG, triglyceride; UA, uric acid.

The above results demonstrated that the gut microbiota plays a mediating role in improving CMFs through different interventions. To further explore the association between individual different responses to interventions and baseline gut microbiota composition, we classified participants into responders and nonresponders based on whether their improvement exceeded the mean change in the control group. Responders showed significantly greater improvements compared with nonresponders following the intervention. Additionally, we plotted receiver operating characteristic (ROC) curves based on baseline gut microbiota composition and found that the area under the curve (AUC) effectively predicted individual responsiveness to different interventions using random forest models (Figure 5). Finally, we identified predictive genera by selecting those that differed between responders and nonresponders among the top 10 genera contributing most to the model (Tables S3 and S4) for different CMFs and validated by *Spearman* correlation.

For weight loss, the AUC were 0.74 (0.40–1.00), 0.90 (0.75–1.00), and 0.82 (0.62–1.00) for RS, FR, and FR–RS group, respectively (Table S5). Additionally, we observed that the baseline relative abundance of four different genera among the top 10 genera ranked by their contribution to the model. Specifically, *Escherichia‐Shigella* had a higher relative abundance in responders, while *Prevotella_9*, *Alistipes*, and *Romboutsia* had lower relative abundances at baseline (Figure 5A). Similarly, for WC reduction, *Alistipes*, and *Romboutsia* showed lower relative abundances in responders after the FR–RS intervention (Figure 5B). However, *Romboutsia* showed higher relative abundances in responders in the FR intervention. For BMI reduction, a total of four genera showed differences between responders and nonresponders in the FR and RS groups, with all of them having higher baseline relative abundance in responders. In FR–RS group, responders exhibited a lower baseline relative abundance in *Alistipes* and *Romboutsia* (Figure 5E).

The baseline microbiota composition exhibited strong predictive capability in reducing body fat. For BFM reduction, the AUCs for the FR, RS, and FR–RS groups were 0.74, 0.71, and 0.81, respectively. In the FR group, *Bacteroides*, *Prevotella_9*, *Coprococcus_1*, and *Subdoligranulum* exhibited lower abundance in responders. In the RS group, *Klebsiella* showed higher abundance in responders. Within the FR–RS group, *Alistipes* exhibited a lower abundance in responders (Figure 5C). For BFP reduction, responders in the FR group showed lower relative abundances of *Bacteroides* and *Subdoligranulum*, and higher abundance of *Romboutsia*. In RS group, lower relative abundances were observed for *Lachnospiraceae_ND3007_group* and *Faecalibacterium*, as well as in FR–RS group for *Dialister* in responders (Figure 5D). Similarly, the ROC curves showed reasonable predictive ability for reducing UA levels (Figure 5K). Only *Roseburia* had a higher baseline relative abundance in responders of FR group for UA reduction (Figure 5F), as well as lower *Parabacteroides* in FR–RS group concerning TG improvement (Figure 5G).

In addition to the random forest model, we explored the logistic regression to assess the predictive ability of baseline gut microbiota. The results showed that the logistic regression models consistently underperformed across all metrics compared with the random forest model (Figure S9). This discrepancy may be due to logistic regression's limitation in handling only linear relationships, while random forests can capture both nonlinear and complex interactions. As a result, we focused our subsequent analyses primarily on the random forest model outcomes.

Meanwhile, the sensitivity analysis revealed no significant changes in the AUC values of the random forest model for different CMFs following the perturbation (Table S6). The feature importance rankings remained consistent, indicating that the model's predictions are robust to minor variations in the data.

**FIGURE 6:**
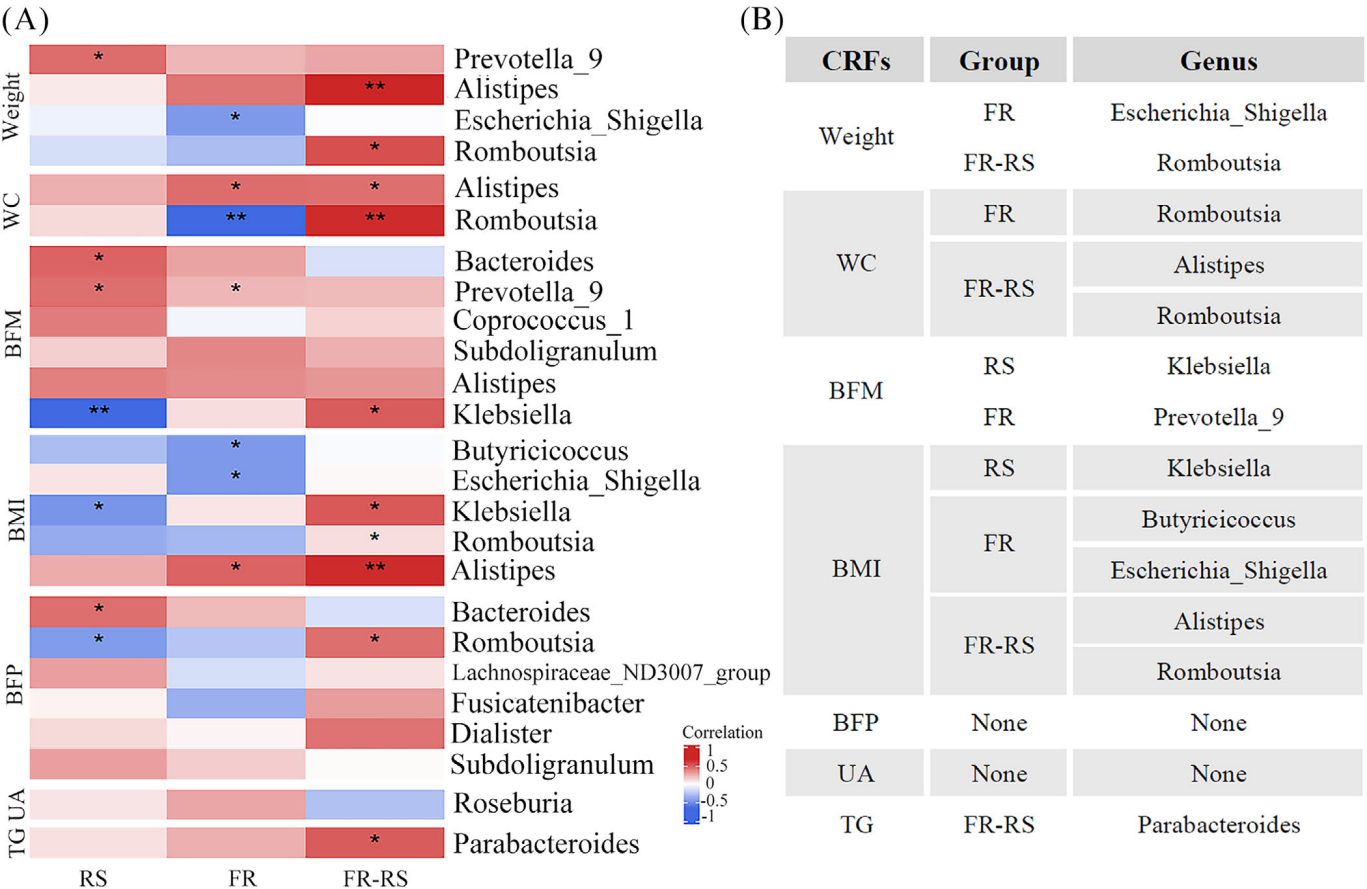
The correlation analysis for predicting CMFs improvement through baseline gut microbiota. (A) Heatmap of the Spearman's correlation coefficients between CMF changes and the relative abundance of baseline gut microbiota. Statistically significant coefficients are marked by * which means *p* < 0.05. (B) Microbial genera associated with changes in specific CMFs and demonstrating excellent predictive capacity for improvement. RS, rope‐kipping group; FR, fiber‐rich group; FR–RS, fiber‐rich diet and rope‐kipping group; CMFs, cardiometabolic factors; WC, waist circumference; BFM, body fat mass; BFP, body fat percentage; BMI, body mass index; TG, triglyceride; UA, uric acid.

To further validate the association between the differential predictive genera and the corresponding CMFs, we conducted a correlation analysis (Figure 6A). The results showed that only a subset of these genera exhibited significant correlations with CMFs (Figure 6B). Specially, we deduce that individuals with higher baseline *Escherichia‐Shigella* abundance experienced better weight improvement with FR intervention, while those with lower baseline *Romboutsia* abundance had greater weight reduction with FR–RS intervention. For interventions targeting WC reduction, individuals with lower baseline *Alistipes* and *Romboutsia* abundance responded more favorable to FR–RS intervention. In terms of BMI reduction, individuals with higher baseline *Butyricicoccus* and *Escherichia‐Shigella* abundance showed enhanced beneficial responsiveness to FR intervention. Whereas those with higher *Klebsiella* abundance experienced better improvement in RS intervention and lower *Alistipes* and *Romboutsia* indicated favorable responses to FR–RS intervention.

When focusing on BFM reduction, lower *Prevotella_9* abundance was associated with improved effectiveness of the FR intervention. The RS intervention was more effective for individuals with lower baseline *Klebsiella* abundance. Finally, individuals with lower baseline *Parabacteroides* abundance experienced improved outcomes with the FR–RS intervention targeting TG levels (all *p* < 0.05) (Figure 6A). Regrettably, although differences in predictive genera between responders and nonresponders for BFP and UA observed (Figure 5D,F), the associations between these genera and changes in BFP or UA were not statistically significant in the correlation analyses (Figure 6A).

## 3 DISCUSSION

In this RCT among undergraduates with overweight/obesity, single interventions were as effective as combined interventions in improving weight, WC, body fat and lipid profile compared with control group at population level. Meanwhile, the FR group further improved LDL‐C and UA. Notably, the responsiveness to FR, RS, or FR–RS interventions on multiple CMFs was person‐specific. Sequential mediation analysis suggested that the effects of different interventions were uniquely mediated by the gut microbiome–metabolite–host axis in improving CMFs. Finally, through random forest analysis of baseline gut microbial features, we found that intervention‐specific baseline microbial profiles could reliably predict individual responsiveness to CMF improvements, aiding in the selection of personalized intervention strategies.

Our findings were consistent with previous studies, demonstrating the FR diet and exercise interventions benefit CMFs improving. For example. a multicenter RCT involving 772 elder participants found a positive correlation between higher fiber intake and reductions in weight and WC.^19^ Similarly, the RS intervention also lead to significant reduction in weight and body fat in obese adults.^20^ At the population level, the results of the three interventions showed that the combined intervention group did not outperform the single intervention groups, which seems to contradict traditional beliefs. This may attribute to the health status of young participants. Younger individuals exhibit a more robust metabolism and greater compensatory capacities compared with the elderly.^21^ Therefore, a single effective intervention may have already reached the maximum limit of improvement in CMFs. Additionally, various lifestyles and environmental factors among young individuals, such as sleep duration and stress levels, could have influence their overall health. In this study, we did not adjust for these confounding factors, and future research is required to elucidate the underlying causes. At the individual level, not all participants responded similarly to the same intervention. This inconsistency may be attributed to individual variations in gut microbiota and their differential responses to the interventions.

The gut microbiota is known to plays a key role in improving CMFs through FR diet and AE.^22^ After the intervention, α and β diversity of participants significantly increased, consistent with previous studies that link higher gut microbiota diversity to improved CMFs.^23^ Beyond the overall structure, we also examined the effects of each intervention on individual bacterial genera. Follow the FR intervention, the relative abundance of *Turicibacter* increased. As a bacterial genus associated with obesity, *Turicibacter* has been linked to reductions in BFP and BFM by lowering TG levels and regulating lipid composition.^24^, ^25^, ^26^ When focusing on body composition improvement, interesting results were noted postintervention. There was a noted increase in *Catenibacterium* relative abundance in the RS group, which is typically found in higher proportions in young obese individuals.^27^ Another RCT involving 106 obese patients concluded that individuals engaging in exercise exhibited a higher proportion of *Catenibacterium*.^28^ However, these findings are not in conflict. The increased presence of *Catenibacterium* in both the exercise and obese groups may indicate different metabolic demands, including its role in regulating the production of SCFAs, polysaccharide breakdown, and other complex metabolic pathways.^29^ The FR–RS intervention effectively increased the abundance of *Ruminococcaceae_UCG‐013*, a butyric acid‐producing bacterium.^30^ Previous research on diet and exercise interventions in obese children indicated that *Ruminococcaceae_UCG‐013* plays a significant role in promoting weight loss.^31^ This may be due to its positive impact on improving lipid metabolism.^32^, ^33^ However, there are currently no studies exploring the association between *Ruminococcaceae_UCG‐013* and UA levels, and further evidence is required to validate our findings.

In the sequential mediation analysis, we identified four “interventions–gut microbiota–metabolites–host” axes. First, in the RS group, the relative abundance of *Holdemanella* increased after the intervention. Research indicates that *Holdemanell*a is negatively correlated with lipid metabolism, implying that it may reduce lipid accumulation by modulating fatty acid‐related metabolic pathways, leading to a decrease in LysoPE levels. As an important molecule in lipid metabolism, LysoPE has been shown in animal studies to reduce body weight by promoting fat metabolism.^34^ Second, in the FR group, the relative abundance of *Turicibacter* increased. Although no studies have yet identified a direct association between *Turicibacter* and l‐asparagine, several studies have confirmed that *Turcibacter* is beneficial for improving hyperlipidemia, with its relative abundance being negatively correlated with LDL‐C levels.^35^, ^36^ At the same time, a study on dietary amino acid patterns found that a diet rich in amino acids such as aspartic acid, arginine, and glycine are associated with lower LDL‐C levels. Aspartic acid it may indirectly contribute to LDL‐C reduction by enhancing overall metabolic health and lipid metabolism as part of comprehensive dietary pattern.^37^

Finally, although current research on *Ruminococcaceae‐UCG‐013* and its relationship with UA and related metabolic intermediates is limited, we can reasonably speculate based on the mediation analysis results. Following the FR–RS intervention, the relative abundance of *Ruminococcaceae‐UCG‐013* increased. Metabolites known for their antioxidant and anti‐inflammatory properties, may help reduce systemic inflammation. Since inflammation is a key factor in elevated UA production, *Ruminococcaceae‐UCG‐013* may indirectly influence UA levels through the anti‐inflammatory actions of its metabolites. While the mediation analysis reveals statistical associations between gut microbiota, metabolites, and CMFs, further research is necessary to confirm these mechanisms.

Given that individuals possess a distinct gut microbiome, it is not unexpected that this microbiome contributes to the diversity of disease manifestations and responses to interventions.^38^ The stronger predictive performance for body weight, body fat, and WC is likely due to their close relationship with energy metabolism and fat storage,^39^ processes that are directly influenced by the gut microbiota through the regulating energy absorption, fat breakdown, and SCFA production. As a result, the model is more effectively distinguish individuals with better or worse outcomes following interventions. In contrast, the weaker predictive performance for TG and UA be attributed to the fact that these outcomes are influenced by more complex factors, including liver and kidney function, as well as genetic predispositions. TG levels are primarily affected by lipid metabolism and dietary fat intake, while UA is regulated by purine metabolism, where the role of gut microbiota is more indirect.^40^ Therefore, the model's predictions for these outcomes are less accurate compared with fat‐ and weight‐related measures. Additionally, individual differences, such as genetics and prior dietary habits, may also contribute to these variations in predictive performance.

Numerous RCT studies also has shown that changes in disease outcomes can be predicted by the gut microbiome.^41^, ^42^ Our results showed that participants with a higher relative abundance of *Escherichia‐Shigella* experienced greater reductions in both weight and BMI after the FR intervention. This could be attributed to the association between higher abundance of *Escherichia‐Shigella* and abnormalities in lipid metabolism.^43^ Furthermore, among individuals with lower baseline levels of *Alistipes*. As a producer of acetic acid and propionic acid, a decrease in *Alistipes* abundance may lead to lower levels of SCFAs, which in turn could exacerbate inflammation, atherosclerosis, and adverse metabolic responses.^44^ Therefore, *Alistipes* may serve as a biomarker for guiding intervention strategies in populations with abdominal obesity and overweight. Last, our research revealed that individuals with high *Parabacteroides* abundance showed better TG reduction effects after the FR–RS intervention. Evidence from animal experiments suggests that *Parabacteroides* has a significant positive impact on obesity in obese mice, which may be attributed to its extensive bile acid conversion functions.^45^ Our study has several limitations. Only a subset of participants was included in omics analysis. However, this is consistent with the sample sizes commonly used in similar studies,^46^, ^47^ and the sample sizes were reasonable. Additionally, as the trial was not originally designed to predict response efficiency based on personalized microbiota composition, our findings require future validation through independent intervention trials. Finally, the relatively small sample size, especially in the serum metabolite analysis, resulted in some mediation effects with *p* values between 0.05 and 0.1 as suggestive trends, which warrant cautious interpretation and require further validation in larger cohort studies.

In conclusion, while FR, RS, and FR–RS interventions were all effective in improving body composition among overweight/obese youth, the FR intervention alone proved to be more suitable for individuals with high LDL‐C and UA levels. This may be attributed to the unique interactions between gut microbiota, metabolites, and the host. Additionally, the variability in individual responses to CMF improvement could be predicted by participants' baseline gut microbiota profiles. In cases where multiple interventions are effective, specific microbes can serve as biomarkers to guide the selection of the most appropriate intervention strategy for each individual.

## 4 METHODS

### 4.1 Inclusion and ethics

This study was registered with the identifier NCT04834687 on clinicaltrials.gov, and received review and approval from the Ethics Committee of the School of Public Health, Sun Yat‐sen University (Batch No. SYSUSPHEC[2021]044). Ensuring the principles of informed consent, all participants were personally notified about the study details and provided their or their parents’ written consent.

### 4.2 Study design and participants

This RCT was conducted between October 2021 and January 2022 at a university in Guangzhou City, China. Recruitment began in September 2021, and 142 undergraduates were initially enrolled. After screening, 129 undergraduates with overweight/obesity were deemed eligible and randomly assigned to one of four groups: the FR diet group (*n* = 32), the RS group (*n* = 33), the FR–RS group (*n* = 33), and the control group (*n* = 31).

Randomization for the four groups was conducted using a random allocation sequence generated from a random‐numbers table, stratified by weight status (normal/overweight/obesity) and sex (male/female). The random allocation sequence was generated by third‐party staff, who concealed the sequence numbers on pieces of paper inside sequentially numbered, sealed, opaque envelopes to ensure allocation concealment. Participants, in their registration order, opened an envelope and were assigned to a group according to the random number inside, under the supervision of third‐party staff.

As for blinding, participants were to four groups without knowing the specific intervention. After assignation, participants remained in their original groups, ensuring no cross‐contamination. Blinding of researchers was maintained during participants enrolment, randomization, on‐site implementation of measurements, and data analysis by having the relevant researchers blinded to group assignments.

All participants were recruited through online advertisements and screened based on specific inclusion and exclusion criteria. The inclusion criteria were: (1) first‐ or second‐year undergraduates with a BMI ≥ 22 kg/m^2^; (2) no weight fluctuation of more than 5 kg in the 3 months prior to the study; (3) signed the informed consent form. Exclusion criteria included: (1) participation in other weight control programs; (2) suffered from secondary obesity resulting from medication or disease; (3) presence of CVDs such as hypertension or diabetes; (4) severe organ disease or physical disabilities.

A sample size of 28 participants per group was required to detect a 1.5 kg reduction in body weight, with a standard deviation (SD) of 2.0  kg, using 80% power and a two‐sided *α* of 0.05, based on data from a comparable population in an 8‐week RCT.^48^ To account for a potential 10% dropout rate, the final sample size was adjusted to 31 participants, resulting in a total of 124 participants across all four groups.

### 4.3 Intervention

The intervention implemented in this study was a dual‐faceted approach, integrating FR dietary and AE components.

#### 4.3.1 Campus Nutrition Window and FR diet intervention

The Campus Nutrition Window, developed in accordance with the Guide for Nutrition and Health School Construction issued by the Chinese government, played a pivotal role in the dietary intervention (refer to Method 1, Supporting Information and Figure S10 for details). The FR diet intervention involved the provision of FR meal packages, carefully crafted by nutrition experts and chefs based on The Dietary Guidelines for Chinese Residents (2016) and other relevant dietary standards. These meals were customized to meet two energy levels according to individual participant needs and were designed to provide no less than 25 g/day of dietary fiber (Method 2, Supporting Information and Figure S11). The meal packages were served to participants in FR and FR–RS groups three times on weekday, with the menu alternating twice per week to maintain variety and encourage adherence.

To ensure rigorous dietary compliance and effective monitoring, an innovative internet‐based dietary intake recording system was employed (Method 2, Supporting Information and Figure S12). Participants were required to meticulously document their meal consumption, including photographic evidence of meal packages before and after eating, along with records of any food waste or additional food intake. On weekends, participants had the freedom to choose their meals but were encouraged to maintain healthy eating habits and continue recording their intake.

#### 4.3.2 AE intervention

The AE component was an internet‐ and on‐site‐integrated RS intervention. Characterized by easy‐accessible, low‐cost, time‐efficient, and minimal space and equipment requirements, RS is a practical and suitable intervention for implementation on Chinese campuses. Participants in the RS and FR–RS groups engaged in a structured RS regimen, consisting of four weekly sessions of 1000 jumps, divided into sets with brief rest periods (Method 3, Supporting Information). The initial phase involved professional supervision, focusing on proper techniques and safety, supported by online instructional materials. This was followed by a peer‐supervised phase, which was designed to optimize exercise efficiency, ensure safety, and enhance compliance with the physical activity regimen (Figure S13).

### 4.4 Outcomes measurements

The primary outcomes of the study were changes in CMFs, categorized into anthropometric indicators: weight, WC, BFM, BFP, BMI; and blood indicators: lipid profiles including TC, LDL‐C, HDL‐C, TG, inflammatory markers such as hs‐CRP, UA, and blood glucose indicators like FPG and FINS. The secondary outcomes involved examining changes in gut microbiota and serum metabolites.

#### 4.4.1 Anthropometric measurements

Weight, body composition, height, and WC were measured by trained nurses both at baseline and after the 8‐week intervention. Weight, BFM, and BFP were assessed using a Body Composition Tester (Inbody, model: 230). Height was measured with a stadiometer (Shkodak, model: TZG). WC was measured following the standard procedure using a flexible tape. BMI was calculated by dividing the weight in kilograms by the square of the height in meters.

#### 4.4.2 Stool sampling and 16S rRNA gene sequencing

A total of 80 participants, 20 from each group, provided stool samples for gene sequencing. Participants were instructed to use the provided kits to collect approximately 3 g of fecal samples within 72 h following their clinical evaluations at both baseline and postintervention. The samples were then stored at −80°C to preserve genomic integrity. The 16S rRNA gene sequencing process included DNA extraction, PCR amplification, library construction, and high‐throughput sequencing (refer to Method 4, Supporting Information for details).

#### 4.4.3 Blood specimen collection, biochemical measurements, and serum metabolomics profiling

A 5 mL venous blood sample was collected from each participant, immediately refrigerated at −20°C, and subsequently centrifuged. The serum was stored at −80°C until further analysis. Biochemical assessments (refer to Method 5, Supporting Information for details), including FPG, FINS, TG, TC, HDL‐C, LDL‐C, UA, and hs‐CRP, were performed at KingMed Diagnostics Group Co., Ltd.

Metabolomics profiling was conducted on a total of 40 participants, with 10 individuals per group. The profiling process involved converting raw data to mzXML format using ProteoWizard, followed by peak detection and metabolite annotation through an R‐based program utilizing XCMS (see Method 5, Supporting Information for details). This comprehensive analysis, leveraging the BiotreeDB MS2 database, enabled the identification of significant metabolites and provided insights into metabolic pathways influenced by the intervention.

### 4.5 Statistical analyses

The normality of data was assessed using the Shapiro–Wilk test. Based on the data distribution, results were expressed as means ± SD for normally distributed data or as medians (25th, 75th percentiles) for non‐normally distributed data. Baseline group differences were evaluated using Pearson's chi‐squared test for categorical variables and either ANOVA or the Kruskal–Wallis *H*‐test for continuous variables.

Intergroup variations in CMF indicators postintervention were analyzed using analysis of covariance (ANCOVA), with baseline values included as covariates. Significant ANCOVA results were followed by SDIAK posthoc tests for pairwise comparisons. Log‐transformations were applied where necessary. Intragroup pre‐ and postintervention differences were evaluated using paired *t*‐tests or Wilcoxon signed‐rank tests. For pairwise comparisons, the Bonferroni test was used for weight, while FDR corrections were applied for other CMFs due to limited statistical power. A two‐sided *p* value < 0.05 was considered statistically significant.

For gut microbiota analysis, alpha diversity indices (Richness, Chao1) were calculated using USEARCH. Beta diversity was evaluated through principal coordinates analysis based on Bray Curtis distance, following by permutational multivariate analysis of variance. Intragroup bacterial differences pre‐ and postinterventions were identified using paired *t*‐tests or Wilcoxon tests, with FDR adjustment (threshold ≤ 0.05). Intergroup bacterial differences were assessed by comparing changes in each bacterium at both the phylum and genus levels, with significance determined using Bonferroni correction.

Metabolite profiles pre‐ and postintervention were analyzed using OPLS‐DA to determine VIP values, with model significance tested via CV‐ANCOVA (SIMCA 14.1). Intragroup differential metabolites were identified based on VIP values (≥1.00) and *p* values (≤0.05) using paired *t*‐tests or Wilcoxon tests. Intergroup differences in metabolites were assessed by comparing log2 fold changes (log2FC) of peak intensities, with significance determined using Bonferroni correction.

Sequential mediation analyses were conducted using the “mediation” package in R, specifically to exam the roles of gut microbiota and serum metabolites in mediating improvements in CMFs. The intestinal microbiota with significant differences between each intervention group and the control group within each group, as well as the serum metabolites with significant differences compared with the baseline were included. *p* Values of the mediating path “intervention–bacteria–CMFs” less than 0.05 were considered significant to identify precise and robust results, meanwhile *p* values for the mediating path “microbiota–metabolites–CMFs” less than 0.1 were considered a trend toward significance to uncover more insightful findings.

Random forest models and logistic regression models were developed using the microbial genus‐level profiles and the responsiveness data of different CMFs in subjects with FR, RS, or FR–RS interventions. To mitigate overfitting in the random forest models, we employed 100 decision trees and implemented sixfold cross‐validation, repeated four times. In each iteration, the dataset was divided into six subsets, with five subsets used for training and one subset for validation. To minimize noise, we first ranked all features according to their importance. Since our ultimate goal is to select the top 10 genera with significant differences as predictive targets, we included the top 20 features in the random forest model to ensure accurate identification of these key genera.

To further assess the model's generalizability, 30% of the data was reserved as an independent validation set, which was not included in the training process. After completing cross‐validation and hyperparameter tuning, we evaluated the model's performance on the independent validation set to ensure robustness on unseen data. For the logistic regression models, stepwise regression based on the Akaike Information Criterion (AIC) was used for variable selection.

We performed a sensitivity analysis to assess the robustness of the random forest models’ predictions to variations in input features. 1% random perturbation was introduced according to the data distribution to simulate small variations. The sensitivity of the random forest models to these changes was assessed by comparing the AUC values before and after the perturbation.

Finally, we evaluated the performance of models across intervention groups by plotting ROC curves and calculating AUC values. For each group, we calculated the AUC and its 95% confidence interval. All analyses were conducted using R 4.2.1.

## AUTHOR CONTRIBUTIONS

Yueqin Zhou conceived and designed the experiments. Zongyu Lin and Fenglian Huang conducted the clinical trial, enrolment and managed the patients. Zongyu Lin and Tianze Li clean the clinical and multiomics data, performed the data analysis, and visualization. Yanna Zhu, Zhijun Lu, and Tianze Li discussed the data and wrote the manuscript. Miao Wu, Lewei Zhu, Yueqin Zhou, Zhijun Lu, and Wei Peng participated in undergraduates’ recruitment and intervention process. Ying‐An Ming and Fei Gao revised the manuscript. All authors have reviewed and approved the final version of the submission manuscript.

## CONFLICT OF INTEREST STATEMENT

The authors declare no conflict of interest.

## ETHICS STATEMENT

This study was registered with the identifier NCT04834687 on clinicaltrials.gov and received review and approval from the Ethics Committee of the School of Public Health, Sun Yat‐sen University (Batch No. SYSUSPHEC[2021]044).

## Supporting information



Supporting Information

## Data Availability

All the data needed to replicate our study's findings will be fully accessible upon publication. Specifically, the microbiota sequence data will be deposited at the NCBI Sequence Read Archive (PRJNA1162639), and the metabolomics data will be available at MetaboLights (MTBLS11131).
