# Supplementary material for: Comparison of diet and exercise on cardiometabolic factors in young adults with overweight/obesity: multiomics analysis and gut microbiota prediction, a randomized controlled trial
Source: MedComm (2020). 2025 Jan 12;6(1):e70044. doi: 10.1002/mco2.70044 (PMC11725046; doi:10.1002/mco2.70044)
Supplement: Supplementary file 1 — Supporting Information [file MCO2-6-e70044-s001.pdf]

Comparison of diet and exercise on cardiometabolic factors in young adults with overweight/obesity: multiomics analysis and gut microbiota prediction—a randomized controlled trial

Zongyu Lin [1, #], Tianze Li [1, #], Fenglian Huang [1, #], Miao Wu [1], Lewei Zhu [1], Yueqin Zhou [1], Ying-an Ming [2], Zhijun Lu [1], Wei Peng [1], Fei Gao [3], and Yanna Zhu [1, 4, *]

[1] Department of Maternal and Child Health, School of Public Health, Sun Yat-sen University, Guangzhou, 510080, China;

[2] Department of Physical Education, Sun Yat-sen University, Guangzhou, 510080, China;

[3] Agricultural Genomics Institute at Shenzhen, Chinese Academy of Agricultural Sciences, Shenzhen, 518000, China;

[4] Guangdong Provincial Key Laboratory of Food, Nutrition and Health, Guangzhou, 510080, China.

[#] These authors contributed equally: Zongyu Lin, Tianze Li, Fenglian Huang.

Corresponding Author: Yanna Zhu, PhD, Associate Professor. Department of Maternal and Child Health, School of Public Health, Sun Yat-sen University, No.74 Zhongshan Second Rd, Guangzhou, Guangdong Province, 510080, China.

Telephone number: +86 020 87334627.

Email address: zhuyn3@mail.sysu.edu.cn

SUPPLEMENTARY MATERIAL

Comparison of diet and exercise on cardiometabolic factors in young adults with overweight/obesity: multiomics analysis and gut microbiota prediction — a randomized controlled trial

Supplementary Methods

Supplementary method 1. Campus nutrition window.

Supplementary method 2. Preparation of the Fiber-rich meal packages, and internet and on-site integrated dietary intervention.

Supplementary method 3. Internet and on-site integrated exercise intervention.

Supplementary method 4. Stool sampling, fecal microbial DNA extraction, amplification, and 16S rRNA gene sequencing.

Supplementary method 5. Blood specimen collection, biochemical measurements, and serum metabolomics profiling.

Supplementary Tables

Table S1. The dietary intake and physical activity intensity of the participants before and after 8-week intervention.

Table S2. The pairwise comparison of the 8-weeks effects among RS, FR, and FR-RS interventions on CMFs in youth.

Table S3. Top 10 most important features and their importance scores from the original random forest model in intervention groups.

Table S4. Importance scores of all features from the adjusted random forest model to mitigate overfitting in intervention groups.

Table S5. All features and their importance scores from the random forest model in the intervention groups after adjustments to prevent overfitting.

Table S6. The area under the curve of baseline gut microbiota predicting cardiometabolic factors across different intervention groups after sensitivities analysis.

Supplementary Figures

Fig. S1. Flowchart of participants through the study, following CONSORT guidelines.

Fig. S2. Individuals who received same intervention express different response according to the changes in CMFs.

Fig. S3. Fecal microbial α-diversity at OTU level within each group before and after 8-week intervention.

Fig. S4. Changes of relative abundance of gut microbiota at phylum level at baseline and 8-week.

Fig. S5. OPLS-DA score plot at negative ion mode of each group before and after 8-week intervention.

Fig. S6. Effects of Fiber-rich diet and Rope-skipping interventions on serum metabolites at negative ion mode.

Fig. S7 The KEGG pathway enrichment analysis of differential metabolites between different intervention groups and the control group.

Fig. S8 KEGG pathway enrichment and impact analysis of differential metabolites between combined intervention and control groups.

Fig. S9 Baseline gut microbiota predict improvements in Cardiometabolic factors base on logistic regression models.

Fig. S10. The Campus Nutrition Window

Fig. S11. Packing boxes and packed meals prepared for the participants.

Fig. S12. The follow-up of participants involved in dietary intervention groups.

Fig. S13. The aerobic exercise intervention.

Supplementary Methods

Supplementary method 1. Campus nutrition window.

The "Healthy China Action 2019-2030" and "National Nutrition Plan 2017-2030," issued by the State Council of China, explicitly outline goals such as meeting the healthcare needs of the people, promoting balanced diets, and health education. Subsequently, the Ministry of Education, in collaboration with the National Health Commission and four other departments of China, released the "Guidelines for Nutrition and Health School Construction." In these guidelines, it is emphasized that the establishment of nutritionally balanced school cafeterias is necessary. In line with these documents, our project team, in collaboration with the catering group of Sun Yat-sen University, set up the "Campus Nutrition Window" on the ground floor of the Songtao Garden Cafeteria at Sun Yat-sen University in Guangzhou city, which was equipped with a team of skilled chefs and experienced nutritionists, and aimed at offering a balanced, quantitative, low-sodium, and low-oil healthy diet.

The Campus Nutrition Window officially started its trial operation in October 2021, catering to all students in Sun Yat-Sen University. Its distinctive features include: 1) Strict portion control of food items (specified quantities for each meal and clear information on total calorie and macronutrient content); 2) Scientific combination of ingredients with a wide variety (no less than 5 different types of food items daily and no less than 25 different types weekly); 3) Cooking method of reduced salt and oil (daily salt intake limited to less than 5g and oil usage ranging from 25-30g); 4) Offering various types of healthy meal packages to all students in the South Campus (including

general health packages, muscle-building packages, and weight-loss packages, etc.).

Supplementary method 2. Preparation of the Fiber-rich meal packages, and internet and on-site integrated dietary intervention.

Quantitative Fiber-rich (FR) meal packages were designed by project staff and nutritionists in alignment with The Dietary Guidelines for Chinese Residents (2016), the China Food Composition (2nd Edition), CHINESE DIETARY REFERENCE INTAKES (2013 Edition), and prepared by skilled chefs at the Campus Nutrition Window. A daily dietary plan regimen comprised three meal packages, encompassing breakfast, lunch, and dinner, and guaranteed a minimum of 25g/day of dietary fiber. Two sets of dietary menus were alternated twice per week (one menu from Monday to Wednesday and another from Thursday to Friday) in order to reduce participants' aversion and fatigue, thus promoting adherence.

In order to design personalized dietary intervention plans, participants in the FR and combined intervention groups were assigned to well-matched, diverse food sources, and quantified diet plans at different energy levels. The estimated ideal body weight (IBW) of each participant was calculated using the empirical Devine formula, which takes into account the height of each individual. Based on the IBW, the daily Estimated Energy Requirement (EER) for each participant was calculated, defined as 25 kcal/kg (IBW)/day, which has been validated in the practise of reducing body weight and improving metabolic parameters [1]. Subsequently, the 25th percentile (1160kcal/day) and the 75th percentile (1630kcal/day) of the rang of the EERs were selected as two representative levels to categorize the EERs for undergraduates in the FR and FR-RS

groups, thus personalizing the energy provided with the three daily meal packages to the greatest extent. When designing the FR diet, meal packages with different amounts of the same type of ingredients were paired to achieve these two different energy levels, ensuring the set energy needs, macronutrient ratios, and dietary fiber levels (25 g/day) were met. Finally, these meal packages were cooked, weighted quantitatively, packaged, and offered to participants at the Campus Nutrition Widow.

Those meal packages were distinguished based on packing boxes that were marked with various colours and features, allowing participants to select their corresponding meals. During the 8-week intervention period, participants received three meal packages each day following the dietary plan from the Campus Nutrition Window, Monday through Friday.

To maintain quality control, participants were required to submit daily dietary intake records using an internet-based methodology. Before each meal, participants were tasked with photographing the meal boxes and upload them online as a check-in form to record package collection. After dining, participants were instructed to indicate whether they fully consumed their meal packages. Furthermore, they were asked to document the type and amount of any food that were discarded after each meal and consumed between meals. Every evening, the project team staff reviews these reports and provided reminders to participants who showed inconsistent compliance.

During weekends, participants in the FR and the combined interventions groups were given autonomy to choose their meals and dining venues. However, they were encouraged to adhere to a healthy diet and required to document their food consumption

for the sake of consistency and assessment. During the intervention period, participants in the FR and combined intervention groups were instructed to maintain their current exercise regimen, and those in the combined intervention and CON groups were required to adhere to their regular dietary habits.

Reference:

1. Nakajima Y, Sato K, Sudo M, et al. Practical dietary calorie management, body weight control and energy expenditure of diabetic patients in short-term hospitalization. *Journal of atherosclerosis and thrombosis*. Jun 30 2010;17(6):558-67. doi:10.5551/jat.3806

Supplementary method 3. Internet and on-site integrated AE exercise intervention.

Participants in both the rope skipping (RS) group and FR-RS group were instructed to follow an RS exercise regimen four times per week, with 1,000 jumps each session. Each session of RS was divided into sets of 100 jumps with a 20-second rest between bouts. The exercise program was divided into two phases: the professional-supervised phase and the peer-supervised phase, both of which included on-site training and internet-based follow-up.

During the initial three weeks, participants in the professionally supervised phase received coaching on Monday, Wednesday, and Saturday evenings. The instructors not only delivered warm-up instruction but also offered comprehensive training supervision, aimed at aiding participants in mastering standardized RS techniques, thereby enhancing exercise efficiency and mitigating the risk of sports injuries. In tandem with the on-site instruction by physical education instructors, the project team, collaborating with the Department of Physical Education of Sun Yat-sen University, prepared online RS training videos, facilitating self-study for participants.

During to the peer-supervised phase, participants formed groups of three to six. They were given the flexibility to schedule their on-site jumping sessions at their preferred days and times, as a strategy to enhance compliance. Nevertheless, they were instructed to make sure that there was at least a full day (24 hours) between any two sessions. In line with the dietary intervention, participants were required to log their jumping sessions and to note the duration and number of jumps in the online documents. The project team staff reviewed the jumping records in the online document every

Wednesday and Saturday, and sporadically examined the jumping videos from various groups throughout each week.

Each participant received a skipping rope for the RS training. Participants in the RS group were instructed to adhere to their regular dietary habits throughout the intervention. Dietary guidance was provided after completing the final assessment. Both the CON group and the FR group were asked to maintain their regular levels of physical activity during intervention period.

Supplementary method 4. Stool sampling, fecal microbial DNA extraction, amplification, and 16S rRNA gene sequencing.

Upon completion of the physical examination, all participants received a fecal sample collection kit, which included a fecal collection tube, a cotton swab, a label, and personal protective equipment. Within three days after the baseline and post-intervention examinations, all participants were required to collect a 3g sample of their own feces, which was to be delivered immediately to the refrigerator at -20 ℃. The samples were then stored in a refrigerator at -80°C in preparation for future gut microbiota testing.

Due to fund capacity, only the top 20 individuals with the highest BMI at baseline in each group were selected for gut microbiota analysis at baseline and 8-week. In accordance with the instructions provided by the DNA extraction kits, genomic DNA was extracted and then evaluated for integrity and purity through 1% agarose gel electrophoresis. The DNA concentration and purity were further determined using the NanoDropone system. PCR amplification was carried out using genomic DNA as the template, followed by product electrophoresis. Primer selection was informed by the desired sequencing regions and incorporated barcodes and PremixTag (TaKaRa). After comparing PCR product concentrations using GeneTools Analysis Software (Version 4.03.05.0, SynGene), the requisite volumes for each sample were calculated based on the principle of equimolarity. Subsequently, the PCR products were mixed in accordance with these calculations. The E.Z.N.A. Gel Extraction Kit was used to recover these mixed PCR products, with target DNA fragments eluted using TE buffer.

Library construction adhered to the standard protocol of the NEBNext® Ultra™ DNA Library Prep Kit for Illumina. Sequencing was then executed on the HiSeq or MiSeq high-throughput platforms. Raw image data files obtained from the sequencing process were transformed into raw sequencing sequences (Raw Reads) via Base Calling analysis. The results were stored in the FASTQ file format, which encompasses both the sequencing sequence (Reads) information and the corresponding sequencing quality information.

In terms of 16s rRNA sequencing, raw paired-end FASTQ files generated from the DNA extraction, PCR amplification, library construction, and sequencing stages were initially filtered using FASTP (version 0.14.1). The sliding window for quality trimming was set at -W 4 -M 20. Primers were removed using CUTADAPT (version 1.14), which is based on the sequence information from both ends, yielding quality-controlled paired-end clean reads. The clean reads were subsequently assembled based on their overlap using the -fastq mergepairs function in USEARCH (version 10), with default settings requiring a minimum overlap length of 16bp and a maximum mismatch of 5bp in the overlapping area. Assembled sequences meeting these criteria were retained and underwent a second round of sliding window quality trimming with FASTP (-W 4 -M 20), providing effective clean assembled sequences. Following this, a feature table was generated using the DADA2 denoise procedure following the QIIME 2 (version 2020.11.0) pipeline. The table was aligned to the SILVA database (V.123) using the -sintax function in USEARCH for taxonomy annotation, with a confidence threshold of 0.8. Features annotated as chloroplasts or mitochondria, or

those not annotated as bacteria at the kingdom level, were removed. The sequence feature table was rarefied to the minimum number of sequences within each sample to minimize the influence of sequencing depths on downstream analyses.

Supplementary method 5. Blood specimen collection, biochemical measurements, and serum metabolomics profiling.

All study participants were instructed to fast from 20:00 the evening before both the baseline and final examinations, with the allowance for minimal water intake. Examinations took place in the morning, during which trained clinical nurses collected a 5 ml fasting blood sample from each participant's antecubital vein. The collected blood samples were immediately stored in a refrigerator set at -20°C, followed by a centrifugal separation procedure (3000r/min, 10min). The separated serum samples were subsequently frozen and stored in a -80°C refrigerator, awaiting further biochemical analysis.

Stored venous blood samples from each participant, preserved at -80 °C, were subsequently analyzed for various health parameters. These included: 1) fasting plasma glucose (FPG), fasting insulin (FINS), homeostatic model assessment of insulin resistance (HOMA-IR), and homeostatic model assessment of β-cell function (HOMA-β); 2) serum lipid profiles, comprising triglyceride (TG), total cholesterol (TC), high-density lipoprotein cholesterol (HDL-C), and low-density lipoprotein cholesterol (LDL-C); and 3) uric acid (UA) and high-sensitivity C-reactive protein (hs-CRP). These assessments were conducted by a biomedical analysis company, KingMed Diagnostics Group Co., Ltd., located in Guangzhou, China.

For serum metabolomics profiling, only the top 10 individuals with the highest BMI at baseline in each group were selected for serum metabolomics analysis, due to budget constraints. For the participants examined, 100 μL of serum sample was

transferred into an Eppendorf tube. We then added 400 μL of an extract solution (acetonitrile: methanol = 1: 1, including an isotopically-labelled internal standard mixture). The samples were vortexed for 30 seconds, sonicated for 10 minutes in an ice-water bath, and incubated at -40℃ for an hour to precipitate proteins. Post incubation, the samples were centrifuged at 12,000 rpm (RCF=13800(×g), R= 8.6cm) for 15 minutes at 4 ℃. The resulting supernatant was transferred to a fresh glass vial for subsequent analysis. Quality control (QC) samples were prepared by mixing equal aliquots of the supernatants from all samples. LC-MS/MS analyses were conducted using an UHPLC system (Vanquish, Thermo Fisher Scientific) with a UPLC BEH Amide column (2.1 mm × 100 mm, 1.7 μm) coupled to an Orbitrap Exploris 120 mass spectrometer (Orbitrap MS, Thermo). The mobile phase consisted of 25 mmol/L ammonium acetate and 25 mmol/L ammonium hydroxide in water (pH = 9.75) (A), and acetonitrile (B). The auto-sampler temperature was set at 4 ℃, and the injection volume was 2 μL. The Orbitrap Exploris 120 mass spectrometer was utilized for its capacity to acquire MS/MS spectra on an information-dependent acquisition (IDA) mode under the control of the acquisition software (Xcalibur, Thermo). In this mode, the acquisition software continually evaluates the full scan MS spectrum. The ESI source conditions were set as follows: sheath gas flow rate at 50 Arb, Aux gas flow rate at 15 Arb, capillary temperature at 320 ℃, full MS resolution at 60000, MS/MS resolution at 15000, collision energy at 10/30/60 in NCE mode, and spray Voltage at 3.8 kV (positive) or -3.4 kV (negative). The raw data were transformed into the mzXML format using ProteoWizard and then processed with an in-house program developed in

R, based on XCMS. This program facilitated peak detection, extraction, alignment, and integration. Following this, a metabolite annotation of each peak was carried out using an MS2 database, known as BiotreeDB. The threshold for annotation was set at 0.3.

Supplementary tables

Table S1. The dietary intake and physical activity intensity of the participants before and after 8-week intervention.

Variables	RS (n = 29)	FR (n = 32)	FR-RS (n = 32)	Control (n = 30)	*P*
**Physical activity intensity, METs/min/week**					
Baseline	1693.0 (1035.0, 1910.0)	1639.0 (924.0, 2332.0)	1702.0 (1266.2, 2621.5)	1794.0 (1024.0, 2506.0)	0.560
8-week	2031.0 (1635.0, 3262.0)	1586.0 (1124.0, 2524.0)	2280.0 (1790.0, 2859.5)	1736.0 (1188.0, 3078.0)	0.167
Within_*P*	**0.002** [r]	0.731 [t]	**0.028** [r]	0.328 [r]	
**Sedentary time, hour**					
Baseline	7.5 (6.0, 9.0)	7.0 (5.0, 9.0)	8.5 (6.5, 10.0)	8.0 (6.0, 10.0)	0.700
8-week	7.3 ± 2.7	7.8 ± 2.0	8.1 ± 3.4	8.7 ± 2.3	0.564
Within_*P*	0.099 [t]	0.754 [r]	0.699 [t]	0.626 [t]	
**Protein intake, g/d**					
Baseline	68.7 (55.6, 85.1)	67.8 (55.9, 93.4)	75.0 (56.6, 89.2)	58.8 (50.5, 79.5)	0.294
8-week	58.1 (48.2, 76.2)	77.4 (65.5, 82.6)	74.4 (58.6, 75.0)	59.1 (42.0, 64.8)	**<0.001**
Within_*P*	0.71 [t]	0.844 [t]	0.057 [r]	**0.012** [r]	
**Dietary fat intake, g/d**					
Baseline	57.8 (44.4, 65.6)	68.1 (45.2, 78.9)	70.3 (54.5, 87.8)	63.5 (54.3, 70.2)	0.080
8-week	69.3 ± 15.2	27.9 ± 8.9	21.6 ± 10.7	66.1 ± 12.3	**<0.001**
Within_*P*	0.047 [r]	**<0.001** [t]	**<0.001** [t]	0.563 [r]	
**Carbohydrates intake, g/d**					
Baseline	205.7 (149.9, 246.6)	219.5 (189.6, 271.8)	205.0 (158.2, 269.3)	188.1 (160.5, 236.0)	0.370
8-week	223.7 (181.5, 233.8)	279.2 (211.1, 298.6)	301.1 (211.7, 305.8)	185.7 (166.5, 219.9)	**<0.001**
Within_*P*	0.609 [t]	0.314 [t]	0.311 [r]	0.055 [t]	
**Energy intake, kcal/d**					
Baseline	1601.5 (1293.0, 1846.0)	1704.5 (1391.0, 2160.0)	1755.0 (1536.0, 2219.0)	1648.5 (1385.0, 1801.5)	0.232
8-week	1749.0 (1537.0, 1829.0)	1668.3 (1271.7, 1782.3)	1688.7 (1253.7, 1734.2)	1601.3 (1322.3, 1708.0)	0.373
Within_*P*	0.256 [t]	**0.041** [t]	**0.002** [r]	0.07 [t]	
**Dietary fiber intake, g/d**					
Baseline	8.4 (5.2, 13.6)	9.7 (6.0, 12.8)	7.6 (5.2, 10.0)	6.6 (5.3, 9.6)	0.450
8-week	5.4 (4.5, 7.7)	25.4 (23.1, 26.0)	25.3 (25.3, 25.6)	6.1 (4.6, 9.5)	**<0.001**
Within_*P*	0.155 [r]	**<0.001** [r]	**<0.001** [r]	0.695 [r]	

Data are presented as mean±SD or median (IQR), *P*-values among groups were determined by analysis of covariance or Kruskal-Wallis test.

P-value for the intra-group comparison prior to and following 8-week intervention was derived through either the paired t-test or the paired Wilcoxon test: [t] for paired t-test and [r] for paired Wilcoxon test.

Abbreviation: FR: Fiber-rich diet group; FR-RS: Fiber-rich diet and rope-skipping group; IQR: Interquartile range; METs: Metabolic equivalents of task; RS: Rope-skipping group.

Table S2. The pairwise comparison of the 8-weeks effects among RS, FR, and FR-RS interventions on CMFs in youth

CMFs	RS **(n = 29)** vs. FR **(n = 32)**	FR **(n = 32)** vs. FR-RS **(n = 32)**	RS **(n = 29)** vs. FR-RS **(n = 32)**
Body weight, kg			
P value	1.000	1.000	1.000
WC, cm			
P value	1.000	0.639	1.000
BFM, kg			
P value	1.000	1.000	1.000
BFP, %			
P value	1.000	1.000	1.000
BMI, kg/m2			
P value	1.000	1.000	1.000
TC, mmol/L			
P value	0.410	0.172	1.000
TG, mmol/L			
P value	0.268	1.000	1.000
LDL-C, mmol/L			
P value	0.684	0.111	1.000
HDL-C, mmol/L			
P value	1.000	1.000	1.000
FPG, mmol/L			
P value	1.000	1.000	1.000
FINs, μU/mL			
P value	1.000	1.000	1.000
UA, μmol/L			
P value	0.545	1.000	1.000

Comparison of 8-week effects adjusting for baseline between intervention groups, assessed by Bonferroni corrected t-test:

Abbreviations: BFM: Body fat mass; BMI: Body mass index; CMFs: Cardiometabolic factors; FINS: Fasting insulin; FPG: Fasting plasma glucose; HDL-C: High-density lipoprotein cholesterol; FR: Fiber-rich diet group; FR-RS: Fiber-rich diet and rope-skipping group; LDL-C: Low-density lipoprotein cholesterol; BFP: Body fat percentage; RS: Rope-skipping group; TC: Total cholesterol; TG: Triglyceride; UA: Uric acid; WC: Waist circumference.

Table S3. Top 10 most important features and their importance scores from the original random forest model in intervention groups.

	FR group		RS group		FR-RS group	
**Rank**	**Genus**	**Value**	**Genus**	**Value**	**Genus**	**Value**
			**Weight**			
1	*Escherichia-Shigella*	1.2836	*Unassigned*	0.6932	*Escherichia-Shigella*	1.5231
2	*Alistipes*	0.965	*Romboutsia*	0.6581	*Prevotella_9*	1.4425
3	*Romboutsia*	0.8954	*Agathobacter*	0.6187	*Bifidobacterium*	0.7359
4	*Tyzzerella_3*	0.822	*Alistipes*	0.5607	*Romboutsia*	0.6159
5	*Ruminococcaceae_UCG-013*	0.5152	*Tyzzerella_3*	0.5293	*Unassigned*	0.6151
6	*Bifidobacterium*	0.514	*Streptococcus*	0.4816	*Alistipes*	0.5081
7	*Agathobacter*	0.5058	*Escherichia-Shigella*	0.4735	*Streptococcus*	0.465
8	*Prevotella_9*	0.4284	*Blautia*	0.4412	*Blautia*	0.4307
9	*Megasphaera*	0.3952	*Dorea*	0.4320	*Haemophilus*	0.4066
10	*Subdoligranulum*	0.3717	*Bifidobacterium*	0.3986	*Clostridium_sensu_stricto_1*	0.3800
			**WC**			
1	*Escherichia-Shigella*	0.7744	*Subdoligranulum*	0.8544	*Streptococcus*	1.1994
2	*Subdoligranulum*	0.6211	*Alistipes*	0.6842	*Subdoligranulum*	0.8073
3	*Bacteroides*	0.5143	*Streptococcus*	0.5822	*Alistipes*	0.7341
4	*Streptococcus*	0.5143	*Dorea*	0.5539	*Escherichia-Shigella*	0.7071
5	*Intestinibacter*	0.4824	*Escherichia-Shigella*	0.5062	*Megamonas*	0.6812
6	*Fusicatenibacter*	0.4334	*Coprococcus_3*	0.4707	*Coprococcus_3*	0.6098
7	*Clostridium_sensu_stricto_1*	0.4017	*Ruminococcaceae_UCG-002*	0.4696	*Fusicatenibacter*	0.5592
8	*Megamonas*	0.3884	*Parabacteroides*	0.4569	*Romboutsia*	0.5476
9	*Roseburia*	0.3842	*Agathobacter*	0.4488	*Unassigned*	0.4455
10	*Romboutsia*	0.4237	*Alistipes*	0.3808	*Bacteroides*	0.4048
			**BFM**			
1	*Anaerostipes*	0.7190	*Prevotella_9*	0.8161	*Prevotella_9*	0.799
2	*Subdoligranulum*	0.7161	*Lachnoclostridium*	0.7424	*Subdoligranulum*	0.781
3	*Lachnoclostridium*	0.6506	*Subdoligranulum*	0.7313	*Agathobacter*	0.6698
4	*Prevotella_9*	0.6151	*Anaerostipes*	0.7057	*Alistipes*	0.5634
5	*Alistipes*	0.5037	*Alistipes*	0.4855	*Faecalibacterium*	0.4864
6	*Bacteroides*	0.4781	*Klebsiella*	0.4473	*Coprococcus_1*	0.4629
7	*Escherichia-Shigella*	0.4581	*Bacteroides*	0.399	*Bacteroides*	0.4001
8	*Parabacteroides*	0.3965	*Turicibacter*	0.3909	*Klebsiella*	0.3879
9	*Fusicatenibacter*	0.3631	*Coprococcus_1*	0.3718	*Akkermansia*	0.3875
10	*Butyricicoccus*	0.3552	*Romboutsia*	0.3345	*Escherichia-Shigella*	0.3734

Table S3-continue. Top 10 most important features and their importance scores from the original random forest model in intervention groups.

	FR group		RS group		FR-RS group	
Rank	Genus	Value	Genus	Value	Genus	Value
			**BFP**			
1	*Dialister*	0.8175	*Dialister*	0.6964	*Subdoligranulum*	0.5633
2	*Anaerostipes*	0.5654	*Romboutsia*	0.6132	*Coprococcus_1*	0.43
3	*Klebsiella*	0.4762	*Anaerostipes*	0.5236	*Anaerostipes*	0.4291
4	*Romboutsia*	0.4506	*Lachnospiraceae_ND3007_group*	0.5083	*Dialister*	0.4039
5	*Bacteroides*	0.4196	*uncultured*	0.431	*Bacteroides*	0.4019
6	*Lachnospiraceae_ND3007_group*	0.3846	*Alistipes*	0.3702	*Romboutsia*	0.387
7	*Turicibacter*	0.37	*CAG-56*	0.3566	*Prevotella_9*	0.3838
8	*CAG-56*	0.3575	*Agathobacter*	0.3509	*CAG-56*	0.3375
9	*Haemophilus*	0.3338	*Faecalibacterium*	0.3352	*Butyricicoccus*	0.3308
10	*Agathobacter*	0.3299	*Roseburia*	0.3341	*Akkermansia*	0.316
			**BMI**			
1	*Alistipes*	0.9681	*Alistipes*	0.7706	*Alistipes*	0.9482
2	*Butyricicoccus*	0.9365	*Romboutsia*	0.7362	*Agathobacter*	0.6394
3	*Lachnospiraceae_ND3007_group*	0.852	*Faecalibacterium*	0.7358	*Anaerostipes*	0.6118
4	*Haemophilus*	0.7659	*Subdoligranulum*	0.6692	*Butyricicoccus*	0.5651
5	*Escherichia-Shigella*	0.674	*Butyricicoccus*	0.6246	*Unassigned*	0.5549
6	*Fusicatenibacter*	0.6427	*Klebsiella*	0.6199	*Fusicatenibacter*	0.5529
7	*Romboutsia*	0.6264	*Agathobacter*	0.6096	*Romboutsia*	0.536
8	*Agathobacter*	0.5748	*Escherichia-Shigella*	0.606	*Bifidobacterium*	0.5267
9	*Subdoligranulum*	0.5521	*CAG-56*	0.5971	*Terrisporobacter*	0.5123
10	*Anaerostipes*	0.5443	*Bacteroides*	0.5806	*Erysipelotrichaceae_UCG-003*	0.4971
			**UA**			
1	*Anaerostipes*	1.1448	*Coprococcus_3*	1.2044	*Roseburia*	0.8729
2	*Coprococcus_3*	1.1366	*Erysipelotrichaceae_UCG-003*	0.8484	*Lachnospiraceae_ND3007_group*	0.7775
3	*Erysipelotrichaceae_UCG-003*	0.7115	*Unassigned*	0.7834	*Anaerostipes*	0.7436
4	*Lachnospiraceae_ND3007_group*	0.6438	*Lachnospiraceae_ND3007_group*	0.7824	*Coprococcus_3*	0.7318
5	*Roseburia*	0.6345	*Roseburia*	0.7708	*Klebsiella*	0.6686
6	*Dorea*	0.5342	*Anaerostipes*	0.7516	*Terrisporobacter*	0.5002
7	*Turicibacter*	0.5218	*Romboutsia*	0.5872	*Dialister*	0.4885
8	*Intestinibacter*	0.5062	*Lachnoclostridium*	0.5086	*Haemophilus*	0.4549
9	*Faecalibacterium*	0.5022	*Clostridium_sensu_stricto_1*	0.5008	*Escherichia-Shigella*	0.4259
10	*Haemophilus*	0.4893	*Dorea*	0.4639	*Unassigned*	0.413

Table S3-continue. Top 10 most important features and their importance scores from the original random forest model in intervention groups.

	FR group		RS group		FR-RS group	
**Rank**	**Genus**	**Value**	**Genus**	**Value**	**Genus**	**Value**
			**TG**			
1	*Lachnoclostridium*	0.8743	*Turicibacter*	0.7852	*Parabacteroides*	1.0155
2	*Parabacteroides*	0.8447	*Bacteroides*	0.7246	*Dialister*	0.7887
3	*Turicibacter*	0.807	*Erysipelotrichaceae_UCG-003*	0.5755	*Subdoligranulum*	0.7666
4	*Dialister*	0.7916	*Ruminococcaceae_UCG-013*	0.5731	*Bacteroides*	0.7141
5	*Alistipes*	0.6284	*Fusicatenibacter*	0.5616	*Alistipes*	0.6401
6	*Subdoligranulum*	0.5678	*Bifidobacterium*	0.5296	*Bifidobacterium*	0.6286
7	*Terrisporobacter*	0.561	*Haemophilus*	0.5148	*Roseburia*	0.5514
8	*Klebsiella*	0.5555	*Coprococcus_3*	0.502	*Turicibacter*	0.5146
9	*Agathobacter*	0.5192	*Blautia*	0.4912	*Ruminococcaceae_UCG-013*	0.4947
10	*Romboutsia*	0.4834	*Romboutsia*	0.4872	*Unassigned*	0.4212

Table S4. Importance scores of all features from the adjusted random forest model to mitigate overfitting in intervention groups.

	FR group		RS group		FR-RS group	
**Rank**	**Genus**	**Value**	**Genus**	**Value**	**Genus**	**Value**
			**Weight**			
1	*Alistipes*	1.4016	*Romboutsia*	1.1741	*Unassigned*	1.104
2	*Romboutsia*	1.1854	*Unassigned*	1.1197	*Tyzzerella_3*	1.101
3	*Agathobacter*	1.1552	*Agathobacter*	1.1192	*Agathobacter*	1.0972
4	*Tyzzerella_3*	1.0277	*Streptococcus*	0.8937	*Romboutsia*	1.0857
5	*Streptococcus*	1.0266	*Tyzzerella_3*	0.8078	*Alistipes*	1.0475
6	*Lachnoclostridium*	0.8278	*Escherichia-Shigella*	0.7711	*Streptococcus*	0.9444
7	*Escherichia-Shigella*	0.7385	*Lachnoclostridium*	0.7691	*Escherichia-Shigella*	0.8381
8	*Dialister*	0.6642	*Bifidobacterium*	0.695	*Bifidobacterium*	0.699
9	*Prevotella_9*	0.6412	*Dorea*	0.6594	*Prevotella_9*	0.6755
10	*Bifidobacterium*	0.6401	*Coprococcus_3*	0.6288	*CAG-56*	0.6492
11	*Coprococcus_3*	0.6085	*CAG-56*	0.6279	*Dialister*	0.6217
12	*Butyricicoccus*	0.577	*Blautia*	0.592	*Dorea*	0.6144
13	*Ruminococcus_2*	0.5744	*Dialister*	0.5866	*Blautia*	0.5355
14	*Dorea*	0.5742	*Prevotella_9*	0.5849	*Lachnospiraceae_ND3007_group*	0.4899
15	*Intestinibacter*	0.5038	*Coprococcus_1*	0.5838	*Subdoligranulum*	0.4321
16	*Subdoligranulum*	0.4643	*Lachnospiraceae_ND3007_group*	0.5661	*Intestinibacter*	0.4125
17	*Haemophilus*	0.3499	*Faecalibacterium*	0.5509	*Haemophilus*	0.409
18	*Megasphaera*	0.3448	*Intestinibacter*	0.4738	*Roseburia*	0.371
19	*Ruminococcaceae_UCG-013*	0.3107	*Anaerostipes*	0.3808	*Clostridium_sensu_stricto_1*	0.364
20	*Phascolarctobacterium*	0.1713	*Klebsiella*	0.2024	*Erysipelotrichaceae_UCG-003*	0.2954
			**WC**			
1	*Escherichia-Shigella*	0.7744	*Subdoligranulum*	*1.3284*	*Subdoligranulum*	*1.8196*
2	*Subdoligranulum*	0.6211	*Alistipes*	0.9842	*Alistipes*	*1.397*
3	*Bacteroides*	0.5143	*Streptococcus*	0.887	*Coprococcus_3*	*1.2326*
4	*Streptococcus*	0.5143	*Coprococcus_3*	0.8809	*Parabacteroides*	*0.9104*
5	*Intestinibacter*	0.4824	*Dorea*	0.8787	*Dorea*	*0.9053*
6	*Fusicatenibacter*	0.4334	*Escherichia-Shigella*	0.7755	*Coprococcus_1*	*0.8701*
7	*Clostridium_sensu_stricto_1*	0.4017	*Ruminococcaceae_UCG-002*	0.7443	*Escherichia-Shigella*	*0.828*
8	*Megamonas*	0.3884	*Parabacteroides*	0.7274	*Romboutsia*	*0.6566*
9	*Roseburia*	0.3842	*Anaerostipes*	0.6788	*Anaerostipes*	*0.5977*
10	*Alistipes*	0.3808	*Agathobacter*	0.6234	*Lachnoclostridium*	*0.5965*
11	*Klebsiella*	0.3785	*Coprococcus_1*	0.5971	*Unassigned*	*0.5578*
12	*CAG-56*	0.3703	*Romboutsia*	0.5889	*Fusicatenibacter*	*0.5214*
13	*Blautia*	0.37	*Tyzzerella_3*	0.5559	*Tyzzerella_3*	*0.5121*
14	*Agathobacter*	0.3595	*CAG-56*	0.5317	*Clostridium_sensu_stricto_1*	*0.47*
15	*Coprococcus_3*	0.3544	*Bacteroides*	0.52	*Turicibacter*	*0.4608*
16	*Ruminococcaceae_UCG-002*	0.354	*Fusicatenibacter*	0.5191	*Roseburia*	*0.4171*

17	*Lachnospiraceae_ND3007_group*	0.3531	*Subdoligranulum*	1.3284	*Blautia*	0.3881
18	*Faecalibacterium*	0.3418	*Alistipes*	0.9842	*Megamonas*	0.3313
19	*Prevotella_9*	0.3199	*Streptococcus*	0.887	*Bacteroides*	0.2982
20	*Romboutsia*	0.3149	*Coprococcus_3*	0.8809	*Terrisporobacter*	0.2245
			**BFM**			
1	*Subdoligranulum*	1.3913	*Subdoligranulum*	1.3884	*Subdoligranulum*	1.2359
2	*Lachnoclostridium*	1.179	*Lachnoclostridium*	1.211	*Prevotella_9*	1.0931
3	*Prevotella_9*	0.8882	*Prevotella_9*	0.9384	*Lachnoclostridium*	1.0574
4	*Anaerostipes*	0.8427	*Anaerostipes*	0.9092	*Anaerostipes*	1.045
5	*Klebsiella*	0.8096	*Alistipes*	0.761	*Alistipes*	0.7247
6	*Alistipes*	0.7123	*Klebsiella*	0.7261	*Klebsiella*	0.6672
7	*Coprococcus_1*	0.5349	*Coprococcus_1*	0.5738	*Escherichia-Shigella*	0.6208
8	*Escherichia-Shigella*	0.5132	*Bacteroides*	0.5462	*Faecalibacterium*	0.5739
9	*Parabacteroides*	0.483	*Parabacteroides*	0.5415	*Bacteroides*	0.5522
10	*Bacteroides*	0.4806	*Faecalibacterium*	0.5323	*Dialister*	0.5145
11	*Dorea*	0.47	*Bifidobacterium*	0.5283	*Coprococcus_1*	0.4823
12	*Faecalibacterium*	0.4353	*Escherichia-Shigella*	0.5257	*Butyricicoccus*	0.4539
13	*Turicibacter*	0.4322	*Agathobacter*	0.5037	*Bifidobacterium*	0.4109
14	*Butyricicoccus*	0.394	*Dorea*	0.5003	*Agathobacter*	0.3853
15	*Fusicatenibacter*	0.355	*Romboutsia*	0.4885	*Ruminococcus_2*	0.3106
16	*Streptococcus*	0.3393	*Turicibacter*	0.4842	*Veillonella*	0.3058
17	*Bifidobacterium*	0.3361	*Fusicatenibacter*	0.4484	*Lachnospiraceae_FCS020_group*	0.2175
18	*Roseburia*	0.3262	*Dialister*	0.4434	*Akkermansia*	0.2117
19	*Dialister*	0.2837	*CAG-56*	0.4012	*Tyzzerella_3*	0.2091
20	*Haemophilus*	0.2706	*Roseburia*	0.3274	*Phascolarctobacterium*	0.2059
			**BFP**			
1	*Dialister*	1.465	*Dialister*	1.4032	*Dialister*	1.514
2	*Romboutsia*	1.0875	*Romboutsia*	1.0616	*Romboutsia*	0.9103
3	*Anaerostipes*	0.873	*CAG-56*	0.8243	*CAG-56*	0.6842
4	*CAG-56*	0.8321	*Anaerostipes*	0.8142	*Alistipes*	0.6027
5	*Lachnospiraceae_ND3007_group*	0.8133	*Lachnospiraceae_ND3007_group*	0.7779	*Anaerostipes*	0.5818
6	*Alistipes*	0.7407	*Alistipes*	0.6794	*Faecalibacterium*	0.5431
7	*Subdoligranulum*	0.5285	*Faecalibacterium*	0.5874	*Akkermansia*	0.5244
8	*Akkermansia*	0.522	*Subdoligranulum*	0.5245	*Subdoligranulum*	0.4659
9	*uncultured*	0.4977	*Turicibacter*	0.4949	*Bacteroides*	0.3577
10	*Bacteroides*	0.4939	*uncultured*	0.4517	*Unassigned*	0.3518
11	*Agathobacter*	0.4762	*Unassigned*	0.4478	*Fusicatenibacter*	0.24
12	*Unassigned*	0.4693	*Akkermansia*	0.4423	*Coprococcus_1*	0.2388
13	*Turicibacter*	0.3908	*Roseburia*	0.4422	*Bifidobacterium*	0.2219
14	*Coprococcus_1*	0.3882	*Bacteroides*	0.4281	*Clostridium_sensu_stricto_1*	0.2199
15	*Lachnoclostridium*	0.3733	*Agathobacter*	0.4026	*Ruminococcus_2*	0.219
16	*Ruminococcaceae_UCG-013*	0.3221	*Fusicatenibacter*	0.376	*Parabacteroides*	0.2108
17	*Klebsiella*	0.3204	*Streptococcus*	0.3554	*Klebsiella*	0.1954
18	*Clostridium_sensu_stricto_1*	0.2805	*Intestinibacter*	0.3377	*Butyricicoccus*	0.1692

19	*Erysipelotrichaceae_UCG-003*	0.2586	*Ruminococcus_2*	0.2727	*Prevotella_9*	0.166
20	*Haemophilus*	0.2462	*Coprococcus_3*	0.2554	*Erysipelotrichaceae_UCG-003*	0.1109
			**BMI**			
1	*Alistipes*	1.5166	*Alistipes*	1.5847	*Alistipes*	1.5684
2	*Faecalibacterium*	1.1305	*Faecalibacterium*	1.1354	*Faecalibacterium*	1.2889
3	*Subdoligranulum*	1.1251	*Romboutsia*	1.0696	*Romboutsia*	1.157
4	*Romboutsia*	1.112	*Unassigned*	1.0563	*Escherichia-Shigella*	1.0844
5	*Unassigned*	0.9585	*Agathobacter*	0.9847	*Subdoligranulum*	1.0399
6	*Butyricicoccus*	0.8598	*Klebsiella*	0.9769	*Bacteroides*	1.0119
7	*Escherichia-Shigella*	0.8568	*Subdoligranulum*	0.9679	*Agathobacter*	1.0046
8	*Agathobacter*	0.8493	*Butyricicoccus*	0.872	*Anaerostipes*	0.9548
9	*Anaerostipes*	0.8051	*Bacteroides*	0.85	*Unassigned*	0.8974
10	*Lachnoclostridium*	0.6901	*Anaerostipes*	0.8119	*Butyricicoccus*	0.8906
11	*Haemophilus*	0.5751	*CAG-56*	0.7872	*Haemophilus*	0.6997
12	*Streptococcus*	0.5477	*Escherichia-Shigella*	0.7852	*Erysipelotrichaceae_UCG-003*	0.6269
13	*Bifidobacterium*	0.4579	*Streptococcus*	0.5574	*Coprococcus_3*	0.6198
14	*Erysipelotrichaceae_UCG-003*	0.4097	*Haemophilus*	0.5556	*Bifidobacterium*	0.6019
15	*Prevotella_9*	0.3935	*Bifidobacterium*	0.5409	*Prevotella_9*	0.5184
16	*Dorea*	0.37	*Coprococcus_1*	0.5131	*Collinsella*	0.5011
17	*Lachnospiraceae_ND3007_group*	0.32	*Clostridium_sensu_stricto_1*	0.4803	*Dorea*	0.4169
18	*Fusicatenibacter*	0.2621	*Dialister*	0.4194	*Fusicatenibacter*	0.4086
19	*Turicibacter*	0.2568	*Erysipelotrichaceae_UCG-003*	0.4068	*Blautia*	0.2533
20	*Parabacteroides*	0.1922	*Lachnospiraceae_ND3007_group*	0.3914	*Terrisporobacter*	0.165
			**UA**			
1	*Coprococcus_3*	2.384	*Coprococcus_3*	1.5811	*Coprococcus_3*	3.09149
2	*Lachnospiraceae_ND3007_group*	1.3981	*Erysipelotrichaceae_UCG-003*	1.3209	*Anaerostipes*	1.62598
3	*Roseburia*	1.2238	*Roseburia*	1.2811	*Roseburia*	1.38842
4	*Erysipelotrichaceae_UCG-003*	1.1732	*Lachnospiraceae_ND3007_group*	1.181	*Lachnospiraceae_ND3007_group*	1.28032
5	*Anaerostipes*	1.1556	*Anaerostipes*	1.1277	*Unassigned*	1.05571
6	*Unassigned*	1.1139	*Unassigned*	0.92	*Romboutsia*	0.72403
7	*Intestinibacter*	0.7178	*Dorea*	0.808	*Tyzzerella_3*	0.7118
8	*Dorea*	0.6132	*Romboutsia*	0.7285	*Agathobacter*	0.54005
9	*Tyzzerella_3*	0.601	*Intestinibacter*	0.7229	*Streptococcus*	0.53412
10	*Turicibacter*	0.5463	*Clostridium_sensu_stricto_1*	0.6855	*Haemophilus*	0.53115
11	*Haemophilus*	0.5219	*Tyzzerella_3*	0.6576	*Dialister*	0.52121
12	*Streptococcus*	0.4699	*Turicibacter*	0.6457	*Lachnoclostridium*	0.50053
13	*Agathobacter*	0.4678	*Lachnoclostridium*	0.6157	*Ruminococcaceae_UCG-013*	0.47292
14	*Collinsella*	0.3999	*Streptococcus*	0.5924	*Fusicatenibacter*	0.44285
15	*Dialister*	0.3881	*Haemophilus*	0.5688	*Klebsiella*	0.39012
16	*Klebsiella*	0.379	*Megamonas*	0.544	*Ruminococcus_2*	0.38941
17	*Bacteroides*	0.3722	*Escherichia-Shigella*	0.5288	*Escherichia-Shigella*	0.37265
18	*Ruminococcus_2*	0.3468	*Agathobacter*	0.5196	*Terrisporobacter*	0.24074
19	*Faecalibacterium*	0.3439	*Ruminococcaceae_UCG-013*	0.4971	*Faecalibacterium*	0.21987
20	*Blautia*	0.2602	*Dialister*	0.4884	*Blautia*	0.09807

TG

1	*Turicibacter*	*1.5216*	*Turicibacter*	*1.0555*	*Turicibacter*	1.3509
2	*Streptococcus*	*1.0833*	*Bacteroides*	*0.9397*	*Bacteroides*	1.0195
3	*Bacteroides*	*1.0817*	*Parabacteroides*	*0.9269*	*Streptococcus*	1.0077
4	*Romboutsia*	*0.9815*	*Romboutsia*	*0.7355*	*Ruminococcaceae_UCG-013*	1.0017
5	*Dialister*	*0.9541*	*Erysipelotrichaceae_UCG-003*	*0.7345*	*Parabacteroides*	0.9827
6	*Fusicatenibacter*	*0.9293*	*Coprococcus_3*	*0.729*	*Fusicatenibacter*	0.9705
7	*Ruminococcaceae_UCG-013*	*0.834*	*Fusicatenibacter*	*0.6401*	*Dialister*	0.957
8	*Parabacteroides*	*0.8133*	*Dialister*	*0.6342*	*Romboutsia*	0.9451
9	*Ruminococcus_2*	*0.767*	*Blautia*	*0.6318*	*Blautia*	0.8834
10	*Subdoligranulum*	*0.6979*	*Streptococcus*	*0.588*	*Bifidobacterium*	0.8488
11	*Agathobacter*	*0.636*	*Agathobacter*	*0.5828*	*Roseburia*	0.7962
12	*Alistipes*	*0.6201*	*Haemophilus*	*0.5657*	*Lachnoclostridium*	0.7954
13	*Lachnoclostridium*	*0.6072*	*Bifidobacterium*	*0.565*	*Faecalibacterium*	0.7756
14	*Klebsiella*	*0.6034*	*Ruminococcus_2*	*0.5531*	*Subdoligranulum*	0.7707
15	*Terrisporobacter*	*0.568*	*Faecalibacterium*	*0.5135*	*Klebsiella*	0.745
16	*Dorea*	*0.5633*	*CAG-56*	*0.4842*	*CAG-56*	0.7414
17	*Unassigned*	*0.5451*	*Anaerostipes*	*0.4223*	*Anaerostipes*	0.6819
18	*Butyricicoccus*	*0.5179*	*Klebsiella*	*0.4175*	*Alistipes*	0.6132
19	*Megamonas*	*0.4668*	*Ruminococcaceae_UCG-013*	*0.3965*	*Ruminococcus_2*	0.5934
20	*Intestinibacter*	*0.4457*	*Ruminococcaceae_UCG-002*	*0.2667*	*Unassigned*	0.5712

Table S5. All features and their importance scores from the random forest model in the intervention groups after adjustments to prevent overfitting.

CMFs	RS (n = 20)	FR (n = 20)	FR-RS (n = 20)
Body weight, kg			
AUC (95%CI)	0.74 (0.40-1.00)	0.90 (0.75-0.100)	0.82 (0.62-1.00)
WC, cm			
AUC (95%CI)	0.71 (0.44-0.98)	0.77 (0.50-1.00)	0.67 (0.36-0.98)
BFM, kg			
AUC (95%CI)	0.74 (0.50-0.98)	0.71 (0.47-0.96)	0.81 (0.59-1.00)
BFP, %			
AUC (95%CI)	0.53 (0.10-0.96)	0.82 (0.56-1.00)	0.78 (0.50-1.00)
BMI, kg/m^2			
AUC (95%CI)	0.72 (0.45-0.98)	0.80 (0.58-1.00)	0.78 (0.55-1.00)
UA, μmol/L			
AUC (95%CI)	0.62 (0.33-0.90)	0.67 (0.41-0.94)	0.69 (0.35-1.00)
TG, mmol/L			
AUC (95%CI)	0.50 (0.15-0.85)	0.68 (0.36-1.00)	0.64 (0.36-0.93)

Abbreviations: AUC: Area under the curve; BFM: Body fat mass; BMI: Body mass index; CI: Confidence interval; CMFs: Cardiometabolic factors; FR: Fiber-rich diet group; FR-RS: Fiber-rich diet and rope-skipping group; hs-CRP: High-sensitivity C-reactive protein; LDL-CH: Low-density lipoprotein cholesterol; BFP: Body fat percentage; RS: Rope-skipping group; TC: Total cholesterol; TG: Triglyceride; UA: Uric acid; WC: Waist circumference.

Table S6. The area under the curve of baseline gut microbiota predicting cardiometabolic factors across different intervention groups after sensitivities analysis.

CMFs	RS (n = 20)	FR (n = 20)	FR-RS (n = 20)
Body weight, kg			
AUC (95%CI)	0.74 (0.40-1.00)	0.90 (0.75-0.100)	0.82 (0.62-1.00)
WC, cm			
AUC (95%CI)	0.71 (0.44-0.98)	0.77 (0.50-1.00)	0.67 (0.36-0.98)
BFM, kg			
AUC (95%CI)	0.74 (0.50-0.98)	0.71 (0.47-0.96)	0.81 (0.59-1.00)
BFP, %			
AUC (95%CI)	0.53 (0.10-0.96)	0.82 (0.56-1.00)	0.78 (0.50-1.00)
BMI, kg/m^2			
AUC (95%CI)	0.72 (0.45-0.98)	0.80 (0.58-1.00)	0.78 (0.55-1.00)
UA, μmol/L			
AUC (95%CI)	0.62 (0.33-0.90)	0.67 (0.41-0.94)	0.69 (0.35-1.00)
TG, mmol/L			
AUC (95%CI)	0.50 (0.15-0.85)	0.68 (0.36-1.00)	0.64 (0.36-0.93)

Abbreviations: AUC: Area under the curve; BFM: Body fat mass; BMI: Body mass index; CI: Confidence interval; CMFs: Cardiometabolic factors; FR: Fiber-rich diet group; FR-RS: Fiber-rich diet and rope-skipping group; hs-CRP: High-sensitivity C-reactive protein; LDL-CH: Low-density lipoprotein cholesterol; BFP: Body fat percentage; RS: Rope-skipping group; TC: Total cholesterol; TG: Triglyceride; UA: Uric acid; WC: Waist circumference.

Supplementary Figures

Fig. S1 Flowchart of participants through the study, following CONSORT guidelines.

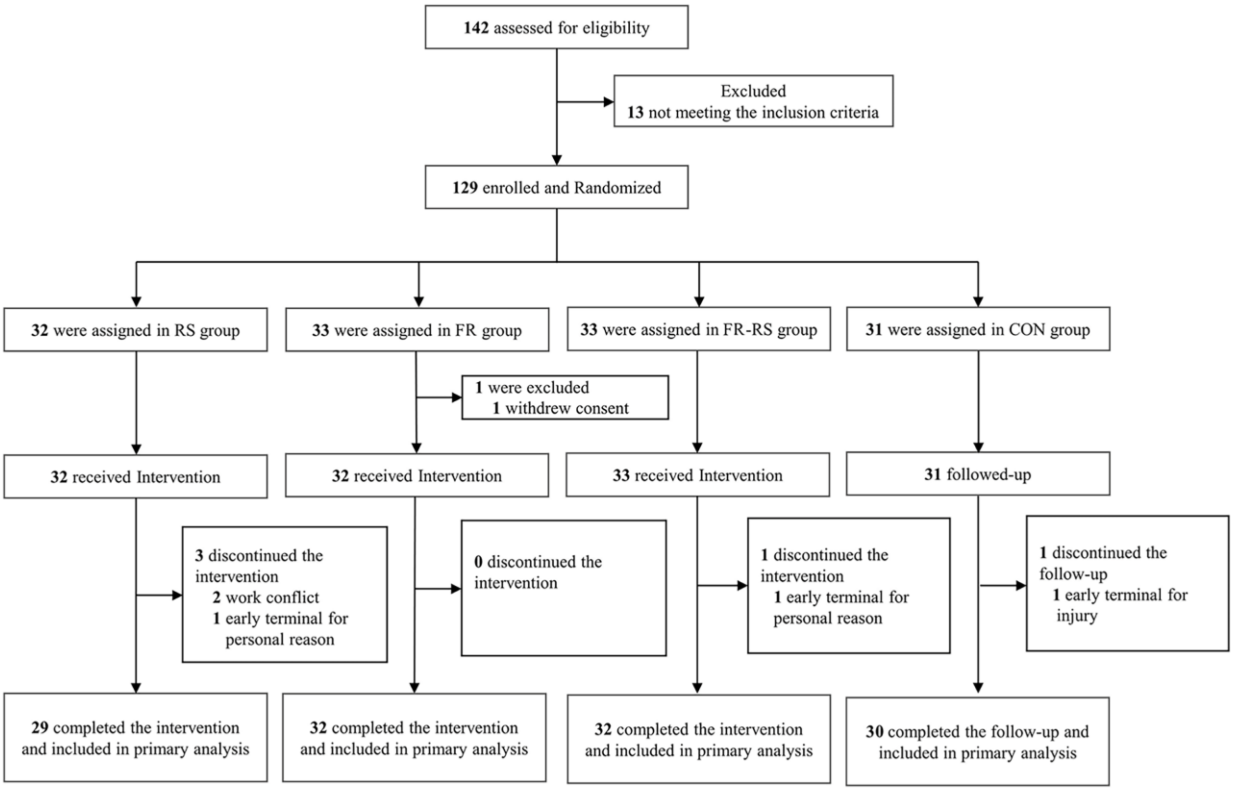

Abbreviations: FR, the Fiber-rich diet group; RS, the rope-skipping group. FR-RS: Fiber-rich diet combined with exercise intervention group.

Fig. S2 Individuals who received same intervention express different response according to the changes in CMFs.

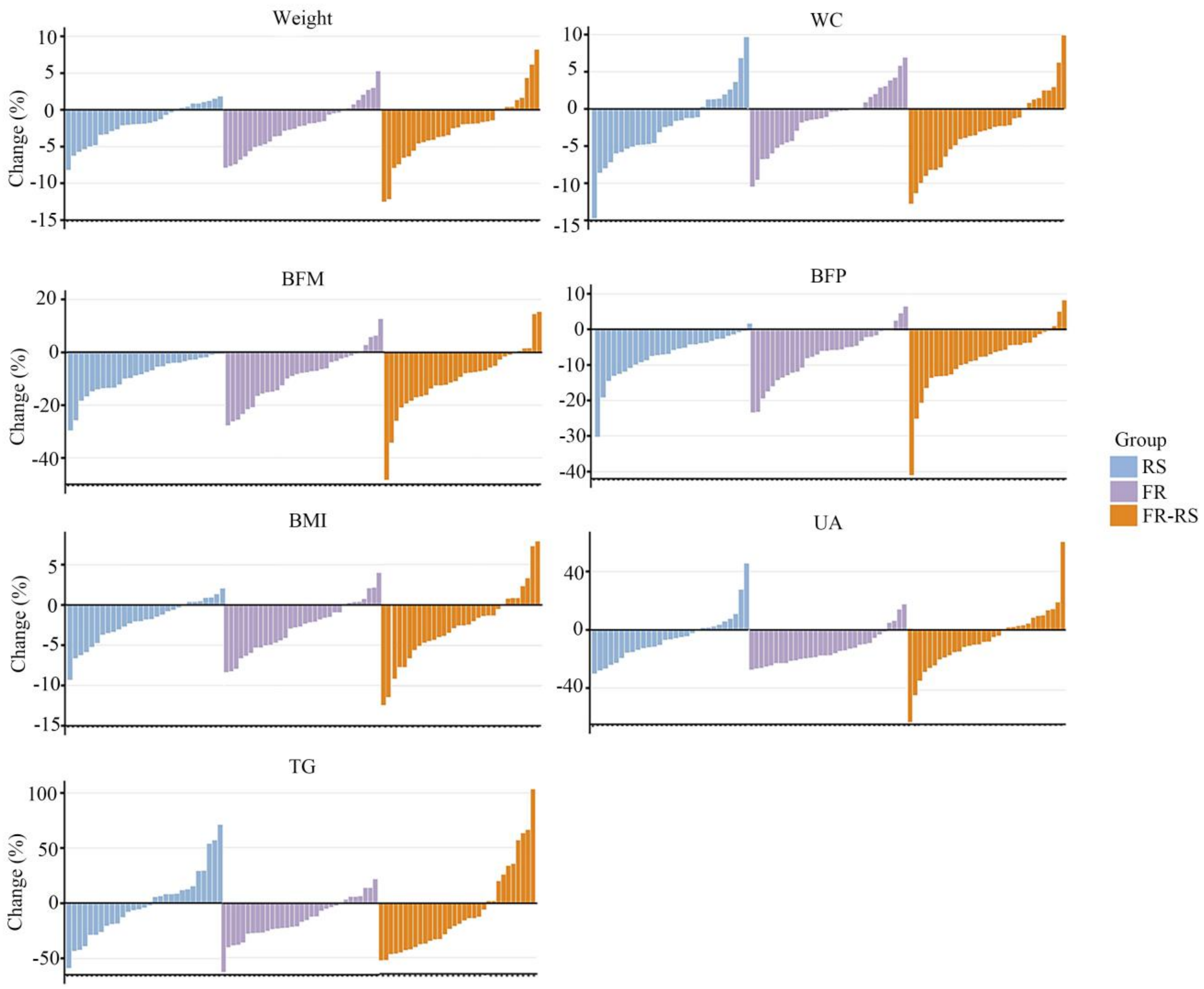

Abbreviations: BFM: Body fat mass; BMI: Body mass index; BFP: Body fat percentage; CMFs: Cardiometabolic factors; LDL-CH: Low-density lipoprotein cholesterol; TC: Total cholesterol; TG: Triglyceride; UA: Uric acid; WC: Waist circumference.

Fig. S3. Fecal microbial α-diversity at OTU level within each group before and after 8-week intervention.

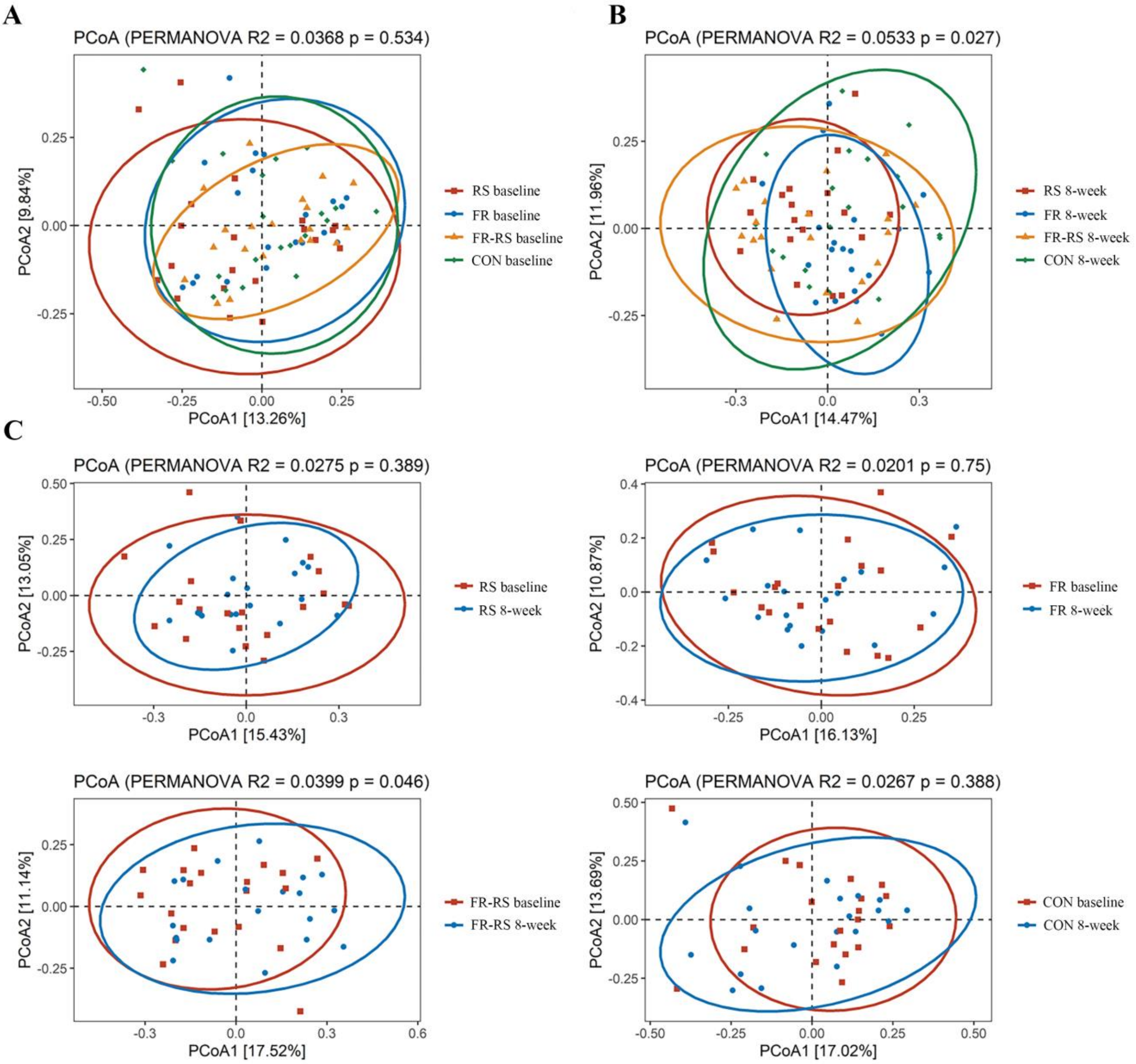

Abbreviations: CON: control group; FR: Fiber-rich diet group; FR-RS: Fiber-rich diet and rope-kipping group. RS: rope-kipping group.

Fig. S4 Changes of relative abundance of gut microbiota at phylum level at baseline and 8-week.

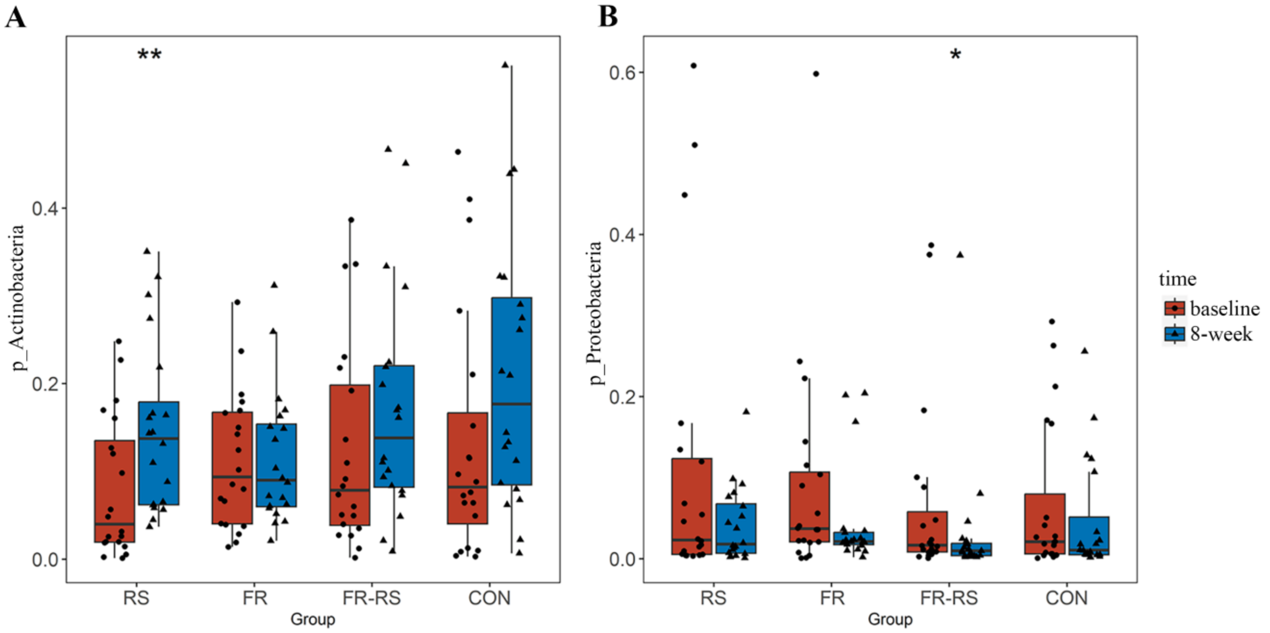

Fig. S4 Changes of relative abundance of gut microbiota at phylum level at baseline (A) and 8-week (B). Within group differences (baseline vs. 8-week) were evaluated by paired t-tests or rank sum tests and corrected for False Discovery Rate: *$P \leq 0.05$, $q\_FDR \leq 0.1$.

Abbreviations: CON: control group; FR: Fiber-rich diet group; FR-RS: Fiber-rich diet and rope-kipping group. RS: rope-kipping group.

Fig. S5 OPLS-DA score plot at negative ion mode of each group before and after 8-week intervention.

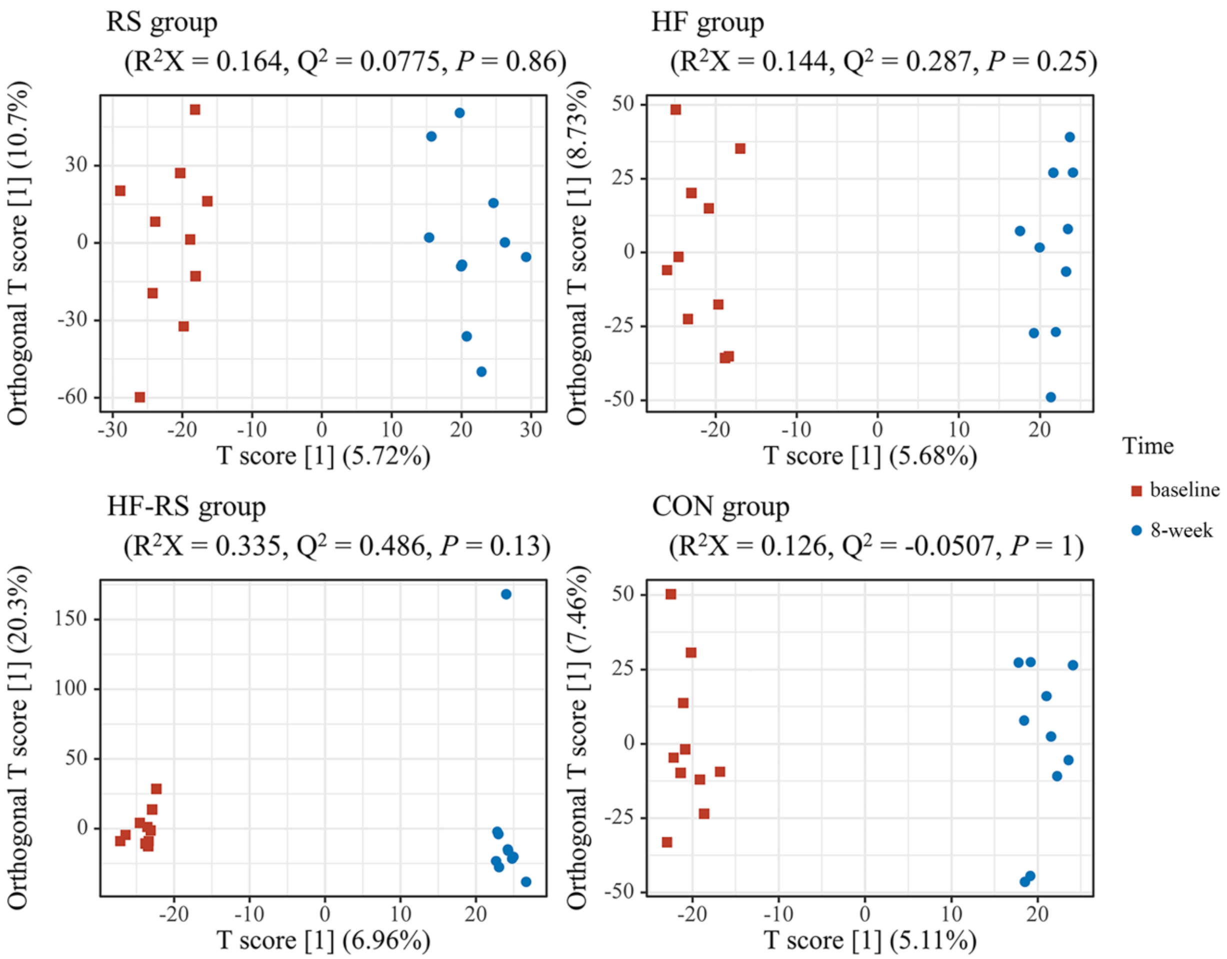

Abbreviations: CON: control group; FR: Fiber-rich diet group; FR-RS: Fiber-rich diet and rope-kipping group. OPLS-DA: Orthogonal Projections to Latent Structures Discriminant Analysis; RS: rope-kipping group.

Fig. S6 Effects of Fiber-rich diet and Rope-skipping interventions on serum metabolites at negative ion mode.

A

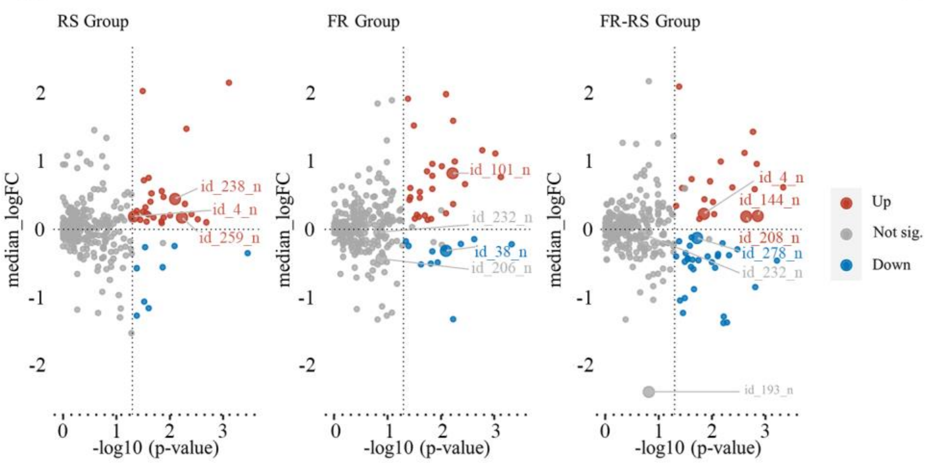

B

Peakid	Metabolites
id_238_n	Purine
id_259_n	Glutathionate(1-)
id_4_n	L-Norleucine
id_101_n	Hydrocortisone
id_206_n	Methylgingerol
id_232_n	Uracil
id_38_n	2-Hydroxystearic acid
id_144_n	L-Phenylalanine
id_193_n	17-phenyl-18,19,20-trinor-prostaglandin E2
id_208_n	trans-Cinnamic acid
id_232_n	Uracil
id_278_n	Picrotoxinin
id_38_n	2-Hydroxystearic acid
id_4_n	L-Norleucine

A: Significantly changed serum metabolites at negative ion mode of each intervention group after 8-week intervention. Colored dots indicate significance referring to baseline. Larger dots with labelled id indicate significance referring to CON group. B: Taxonomy information of labelled metabolite.

Abbreviations: CON: control group; FR: Fiber-rich diet group; FR-RS: Fiber-rich diet and rope-kipping group.

Fig. S7 The KEGG pathway enrichment analysis of differential metabolites between different intervention groups and the control group.

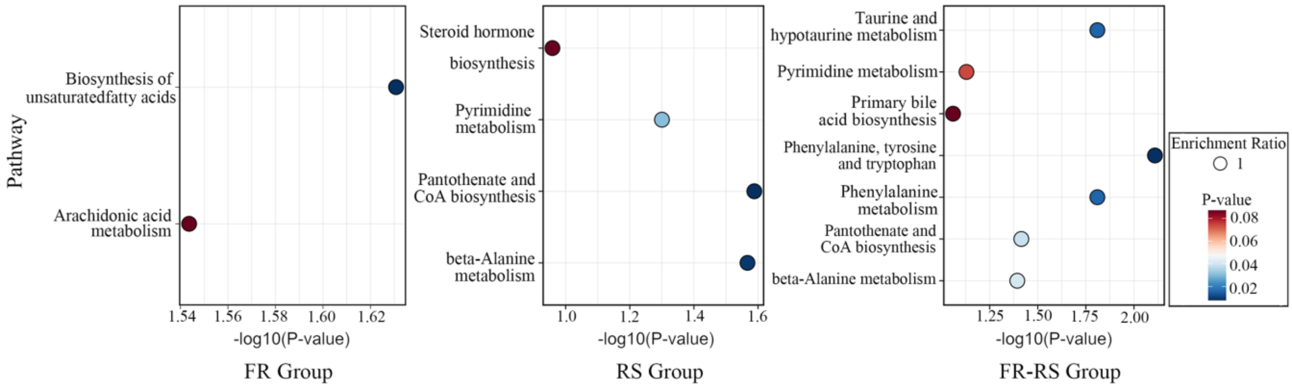

Abbreviations: CON: control group; FR: Fiber-rich diet group; FR-RS: Fiber-rich diet and rope-kipping group.

Fig. S8 KEGG pathway enrichment and impact analysis of differential metabolites between combined intervention and control groups.

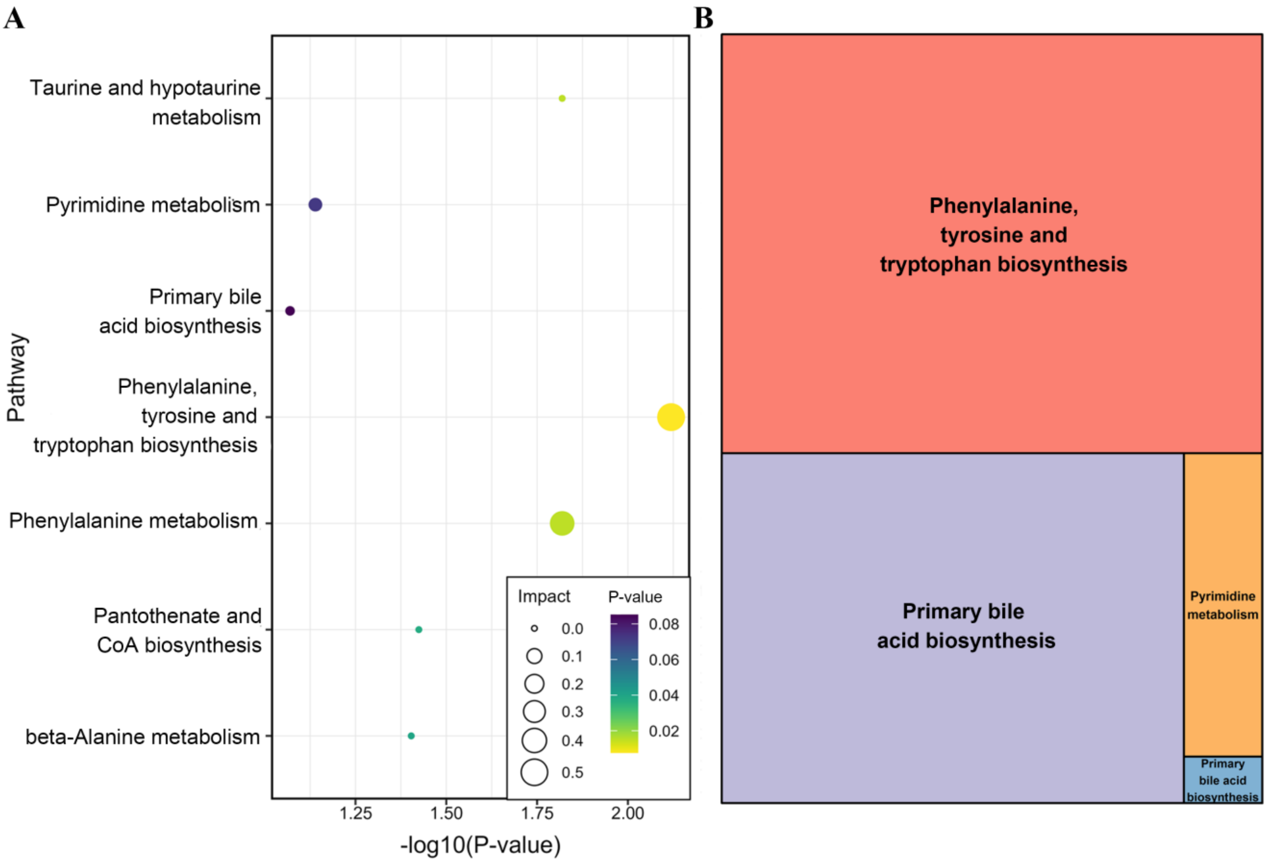

A: KEGG pathway enrichment bubble plot of differential metabolites, B: KEGG pathway impact treemap of differential metabolites.

Fig. S9 Baseline gut microbiota predict improvements in CMFs base on logistic regression models.

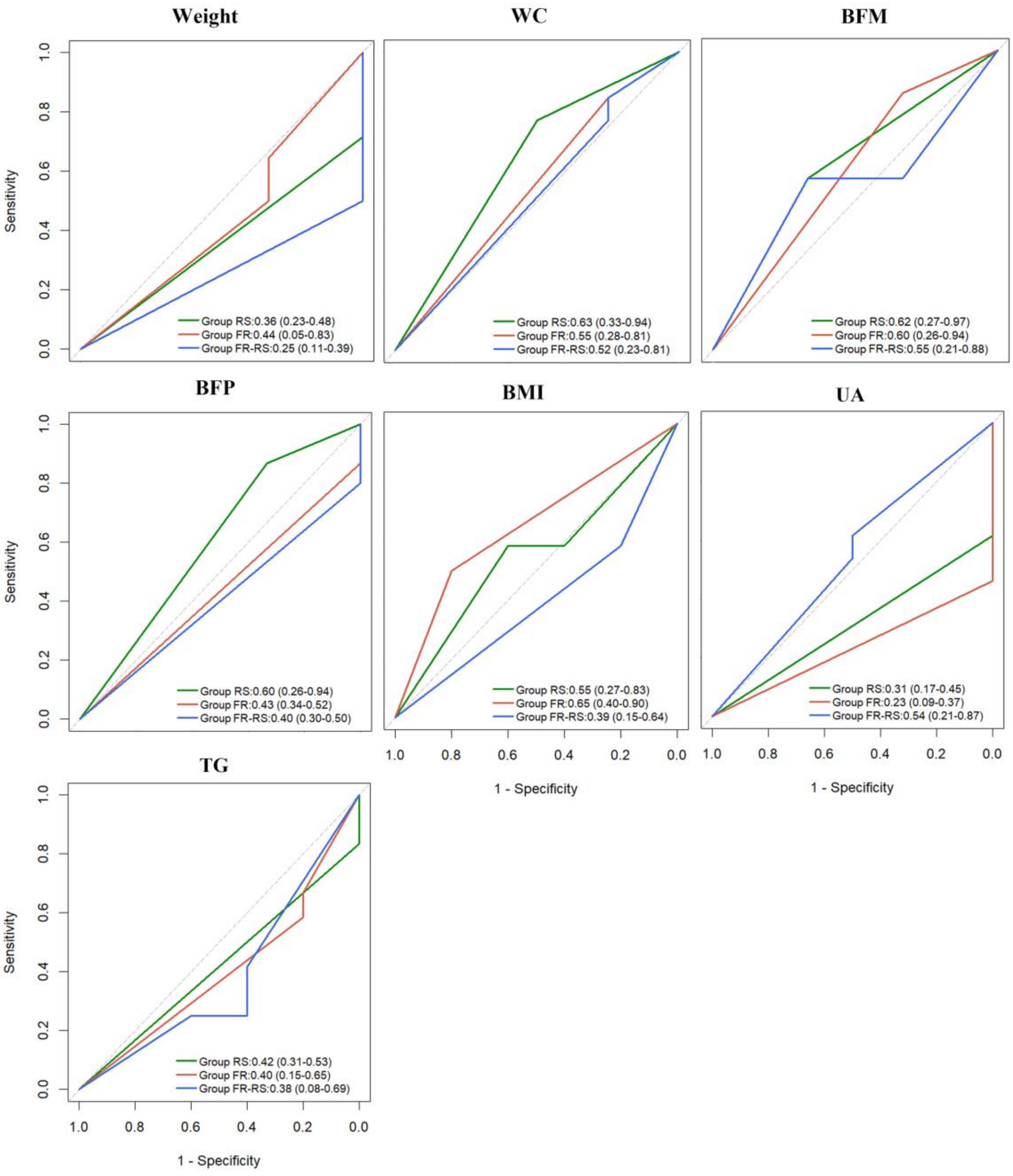

Abbreviations: RS: rope-kipping group; FR: fiber-rich group; FR-RS: fiber-rich diet and rope-kipping group. CMFs: cardiometabolic factors. WC: waist circumference; BFM: Body fat mass; BFP: Body fat percentage; BMI: Body mass index; TG, triglyceride; UA, uric acid.

Fig. S10 The Campus Nutrition Window

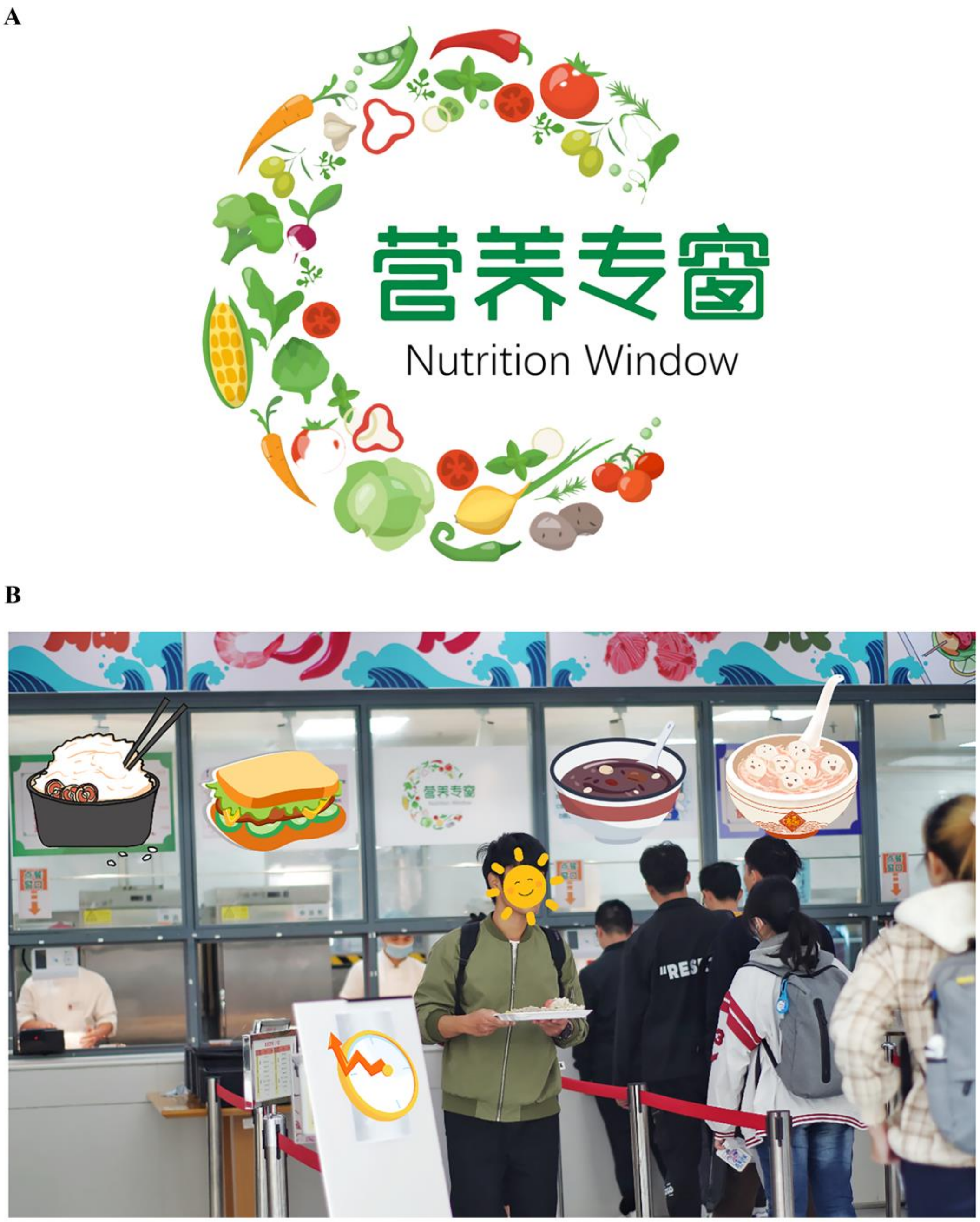

A Slogan of the Campus Nutrition Window. B A scene of college students picking up meals at the Campus Nutrition Window

Fig. S11 Packing boxes and packed meals prepared for the participants.

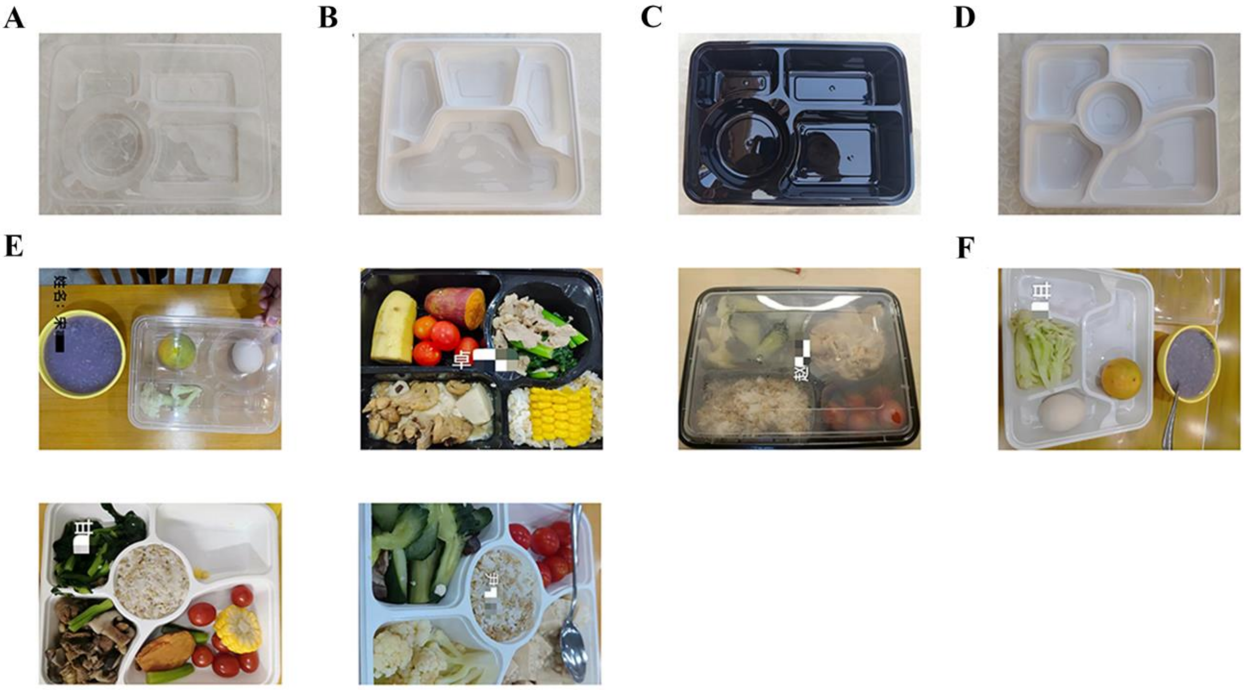

A Transparent four-compartment packing box: Used for packing breakfast for participants with an EER of 1630kcal/d. B White opaque four-compartment packing box: Used for packing breakfast for participants with an EER of 1160kcal/d. C Black opaque four-compartment packing box: Used for packing lunch and dinner for participants with an EER of 1630kcal/d. D White opaque five-compartment packing box: Used for packing lunch and dinner for participants with an EER of 1160kcal/d. E Example of breakfast, lunch, and dinner for participants with an EER of 1630kcal/d. (f) Example of breakfast, lunch, and dinner for participants with an EER of 1160kcal/d. Sample photos were taken by participants after retrieving their meals, with personal information redacted.

Abbreviation: EER: Estimated Energy Requirement.

Fig. S12 The follow-up of participants involved in dietary intervention groups.

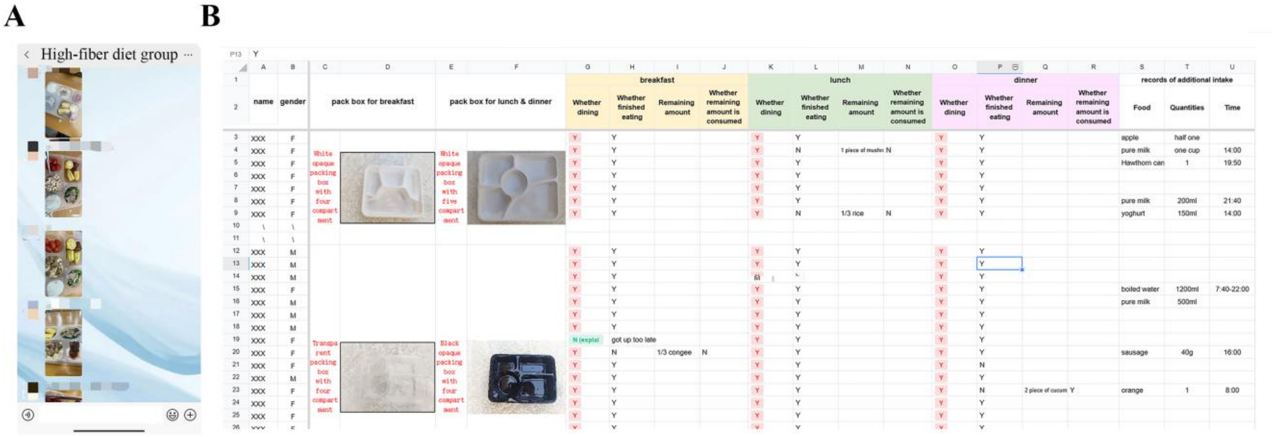

A The meal-packages were taken by participants after retrieving their meals and sent to the WeChat group as a check-in. Personal information was redacted. B Daily dietary records for follow-up participants.

Fig. S13 The aerobic exercise intervention.

A B

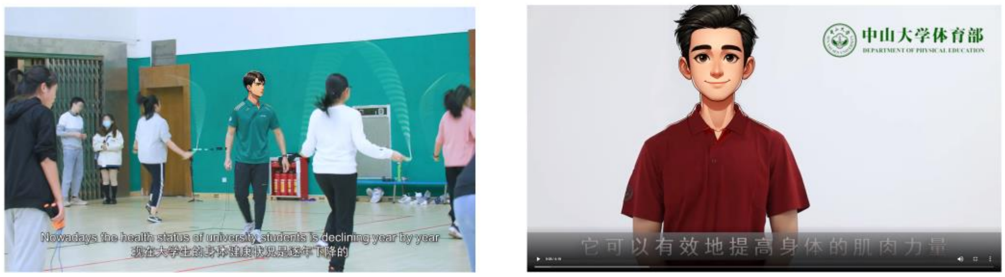

C

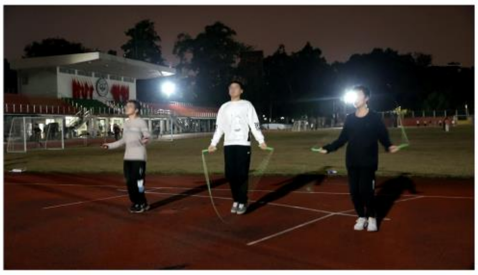

D

Name	Gender	1st jump of this week		2nd jump of this week		3rd jump of this week		4th jump of this week	
		date	times of jump	date	times of jump	date	times of jump	date	times of jump
XXX	F	2021/11/8	1000	2021/11/10	1000	2021/11/12	1000	2021/11/13	1000
XXX	F	2021/11/8	1000	2021/11/10	800	2021/11/12	700	took a leave	
XXX	F	2021/11/11	1000	2021/11/12	1000	2021/11/13	1000	2021/11/14	1000
XXX	M	2021/11/8	850	2021/11/10	800	2021/11/12	1000	2021/11/13	1000
XXX	F	2021/11/8	400	2021/11/10	400	2021/11/12		2021/11/14	1000
XXX	F	2021/11/8	1000	2021/11/10	1000	2021/11/12	1000	2021/11/13	1000
XXX	F	2021/11/8	1000	2021/11/10	1000	2021/11/12	1000	2021/11/13	1000
XXX	F	2021/11/8	1000	2021/11/9	1000	2021/11/11	1000	2021/11/12	1000
XXX	F	2021/11/8	1000	2021/11/10	1000	2021/11/12	1000	2021/11/13	1000
XXX	M	2021/11/8	1000	2021/11/9	1000	took a leave		took a leave	
XXX	F	2021/11/8	1000	2021/11/10	1000	2021/11/12	1000	2021/11/13	600
XXX	F	2021/11/8	1000	2021/11/10	1000	2021/11/12	1000	2021/11/13	1000
XXX	F	2021/11/8	1000	2021/11/10	1000	2021/11/12	1000	2021/11/14	1000
XXX	M	2021/11/8	1000	2021/11/11	1000	2021/11/12	1000	2021/11/13	1000

A: A scene of a professional PE teacher supervising and instructing participants on their rope skipping exercises;B: Scene of online rope skipping instruction video; C: A scene of grouped participants roping skip on the playground; D: The records of follow-up of participants involved in rope-skipping intervention through Online documents.
